# One-Step Aqueous Synthesis of Anionic and Cationic AgInS_2_ Quantum Dots and Their Utility in Improving the Efficacy of ALA-Based Photodynamic Therapy

**DOI:** 10.1021/acs.inorgchem.1c03298

**Published:** 2022-02-01

**Authors:** Mahshid Hashemkhani, Marilena Loizidou, Alexander J. MacRobert, Havva Yagci Acar

**Affiliations:** † Graduate School of Materials Science and Engineering, Koç University, Rumelifeneri Yolu, Sariyer, Istanbul 34450, Turkey; ‡ Division of Surgery and Interventional Science, Centre for Nanomedicine and Surgical Theranostics, University College London, Royal Free Campus, Rowland Hill Street, London NW3 2PE, U.K.; § Department of Chemistry, Koç University, KUYTAM, Rumelifeneri Yolu, Sariyer, Istanbul 34450, Turkey

## Abstract

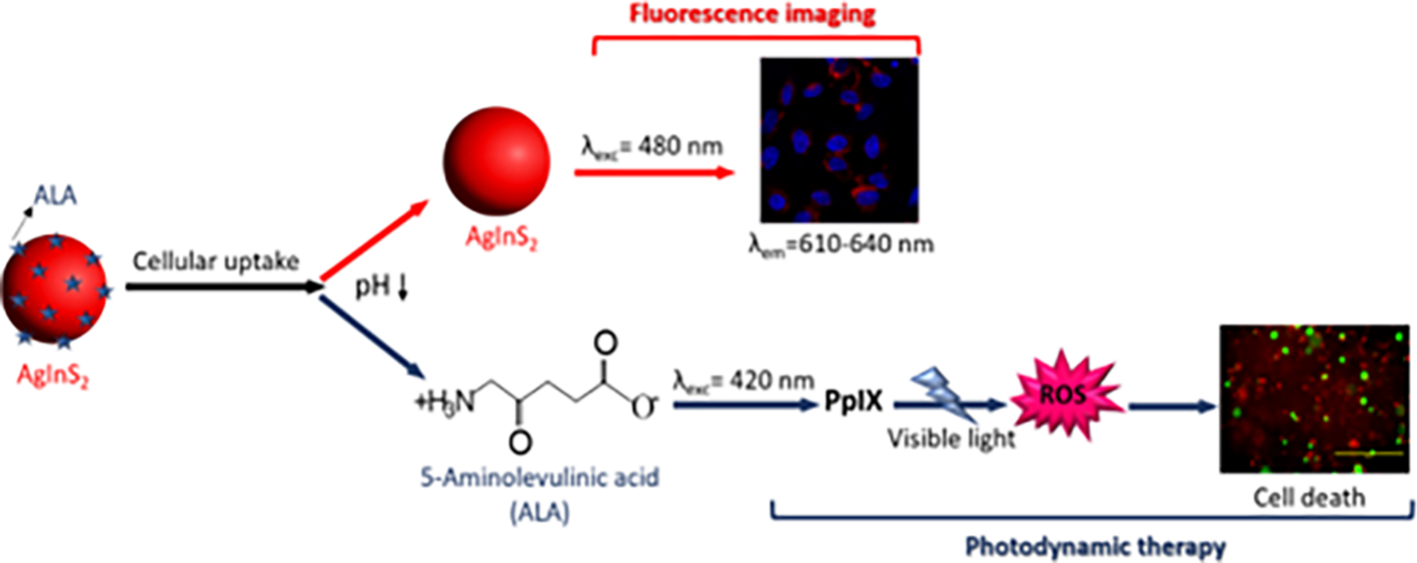

Silver–indium–sulfide quantum dots (AIS QDs) have potential applications in many areas, including biomedicine. Their lack of regulated heavy metals, unlike many commercialized QDs, stands out as an advantage, but the necessity for alloyed or core–shell structures and related costly and sophisticated processes for the production of stable and high quantum yield aqueous AIS QDs are the current challenges. The present study demonstrates the one-step aqueous synthesis of simple AgInS_2_ QD compositions utilizing for the first time either a polyethyleneimine/2-mercaptopropionic acid (AIS-PEI/2MPA) mixture or only 2-mercaptopropionic acid (AIS-2MPA) as the stabilizing molecules, providing a AgInS_2_ portfolio consisting of cationic and anionic AIS QDs, respectively, and tuneable emission. Small AIS QDs with long-term stability and high quantum yields (19–23%) were achieved at a molar ratio of Ag/In/S 1/10/10 in water without any dopant or a semiconductor shell. The theranostic potential of these cationic and anionic AIS QDs was also evaluated in vitro. Non-toxic doses were determined, and fluorescence imaging potential was demonstrated. More importantly, these QDs were electrostatically loaded with zwitterionic 5-aminolevulinic acid (ALA) as a prodrug to enhance the tumor availability of ALA and to improve ALA-induced porphyrin photodynamic therapy (PDT). This is the first study investigating the influence of nanoparticle charge on ALA binding, release, and therapeutic efficacy. Surface charge was found to be more critical in cellular internalization and dark toxicity rather than drug loading and release. Both QDs provided enhanced ALA release at acidic pH but protected the prodrug at physiological pH, which is critical for tumor delivery of ALA, which suffers from low bioavailability. The PDT efficacy of the ALA-loaded AIS QDs was tested in 2D monolayers and 3D constructs of HT29 and SW480 human colon adenocarcinoma cancer cell lines. The incorporation of ALA delivery by the AIS QDs, which on their own do not cause phototoxicity, elicited significant cell death due to enhanced light-induced ROS generation and apoptotic/necrotic cell death, reducing the IC50 for ALA dramatically to about 0.1 and 0.01 mM in anionic and cationic AIS QDs, respectively. Combined with simple synthetic methods, the strong intracellular photoluminescence of AIS QDs, good biocompatibility of especially the anionic AIS QDs, and the ability to act as drug carriers for effective PDT signify that the AIS QDs, in particular AIS-2MPA, are highly promising theranostic QDs.

## Abstract 2

Use of the ALA-loaded cationic and anionic AIS QDs for visible light PDT coupled with QD-based optical imaging in the medical imaging window was studied.

## Introduction

Semiconductor quantum dots (QDs) have attracted great interest as a novel class of promising optical probes in the past two decades.^1,2^ Compared to bulk materials, they exhibit many advantages, such as size-dependent photoluminescence (PL), high PL quantum yields (QY), sharp and symmetrical fluorescence peaks, broad excitation spectra, large Stokes shifts, and long-lasting luminesce, which is valuable for medical imaging.^3,4^ Recently, heavy metal-free ternary I–III–VI_2_ QDs such as CuInS_2_,^5^ CuInSe_2_,^6^ and AgInS_2_^7,8^ have been intensively investigated. AgInS_2_ (AIS) QDs are promising due to their relatively wide bandgaps ranging from 1.87 to 2.03 eV that provide tunable luminescence in the visible region, especially in the optical window from 600 to 800 nm suitable for medical imaging.^6,8,9^ Besides, a more stable nature and synthesis at lower temperatures are important advantages of AIS over CuInS_2_, making it attractive for medical purposes. However, the synthetic procedures for high-quality ternary I–III–VI_2_ QDs usually involve non-polar organic solvents, which might limit the direct applications of these QDs in biological applications.^8−11^ Often, quaternary compositions with Zn or core–shell structures (AIS/ZnS) are preferred to improve the QY and stability.^12−15^ However, this strategy usually causes a blue shift in the emission wavelength due to the wider bandgap of ZnS, which may not always be suitable for applications, e.g., for optical imaging best achieved between 600 and 900 nm. Moreover, the complexity and cost of these organic routes and hydrophobicity of the produced QDs are significant issues in large production and for application in biotechnology and medicine wherein aqueous colloidal particles are needed.

The transfer of the hydrophobic QDs into the aqueous phase is usually achieved by post-synthetic ligand exchange or by phase-transfer using amphiphilic copolymers, which causes a significant drop in QY.^10,11,16^ Alternative aqueous synthetic protocols usually cause low QY, hence almost always, a ZnS shell or Zn–Ag–In–S (ZAIS) quaternary systems are preferred.^7,10,17,18^ Deng et al.^7,17^ produced glutathione (GSH)-coated ZAIS QDs in water with tunable PL in the range of 550–605 nm and QY between 10 and 30% (with respect to R6G). Luo et al.^19^ synthesized GSH-coated AIS nanocrystals in water at 95 °C with a maximum QY of 3%. They investigated the influence of pH, GSH/In, In/Ag, and In/S ratios on the optical properties of AIS. It was shown that pH 8.5 and a high GSH/In ratio could increase the PL intensity (peak position was not changed), whereas the ratios of In/Ag and In/S could affect the emission wavelength and intensity. Recently, we managed to produce stable GSH-coated AIS QDs without Zn, emitting at 634 nm with a 21% QY by applying a cation-rich formulation and avoiding large excess use of GSH (Ag/In/S/GSH 1/4/2.5/10 mole ratio).^3^

Xu et al. applied hydrothermal synthesis to produce cysteine-coated AIS/ZnS with QY of as high as 35% using thioacetamide (TAA) to control the growth rather than the fast sulfur-releasing Na_2_S.^20^ Recently, Mrad et al. reported 3-mercaptopropionic acid (3MPA)-coated AIS QDs with 5.5–15.6% QY and AIS/ZnS with up to 78% QY after fractionation of the products.^8^ The key roles of 3MPA were reported as sulfur release at high temperatures, ability to form a ligand–cation complex with both In^3+^ and Ag^+^, and bidentate binding to the surface, causing passivation of the chalcogenide vacancies. One additional important point would be the lack of emission tunability of AIS unless zinc was added. Regulacio et al. applied polyacrylic acid (PAA) and mercaptoacetic acid (MAA) simultaneously to improve the QY of the aqueous AIS/ZnS QDs to 20%.^21^ However, the use of only MAA or PAA provided very poor luminescence. They suggested that polyacrylic acid would bind In^3+^ and regulate its reactivity, whereas the thiol of MAA would bind Ag^+^. Another critical function of PAA was also reported as enhancing the colloidal stability of these QDs because the aqueous AIS QDs also lack long-term stability.^19,22,23^ Indeed, utilization of the PAA/MAA-mixed coating approach was first reported by our group to improve the colloidal stability of the MAA-coated CdS while maintaining strong luminescence.^24^

To the best of our knowledge, there are only two examples of cationic aqueous AIS QDs. Raevskaya et al.^25^ reported the preparation of branched polyethyleneimine (PEI-25 kDa)-coated AIS QDs with emission maxima at 590 nm and maximum QY of 20% (with respect to anthracene) when a Ag/In/S ratio of 1/5/5 was used but only after 2 h post-synthesis thermal treatment at 100 °C. They have also reported the formation of about 100 nm aggregates. Wang et al.^26^ used high amounts of lower molecular weight PEI (10 kDa) and a pressure cooker (120 °C) to produce cationic AIS QDs (10 kDa) with yellow luminescence (emission maxima at 560 nm) and 32% QY in small hydrodynamic sizes (18.1 nm) using a Ag/In/S mole ratio of 1/16/10. They could not achieve emission wavelength tuning by changing any of the variables. They attributed this to the lack of sulfur-containing ligands, hence many S-vacancies. Therefore, there is still a clear need to develop simple, economical, aqueous synthetic protocols for the preparation of functional, stable, and highly luminescent AIS with emission at and above 600 nm for medical applications. To achieve this, the coating molecules should be selected carefully, and the stoichiometry of the components should be optimized. Here, we do propose 2-mercaptopropionic acid (2MPA) to produce an anionic and PEI (25 kDa)/2MPA mixture to produce cationic AIS QDs with long-term stability, tunability, and strong luminescence. We have previously demonstrated the advantage of using the PEI/2MPA mixture over the PEI coating on the luminescence properties of Ag_2_S QDs and on improving the biocompatibility of the cationic particles. Polymeric coating molecules provide colloidal stability due to multidentate binding to the nanocrystal surface but produce poor luminescence due to the lack of effective surface passivation and surface defects.^24,27,28^ However, if they are mixed with small, thiolated molecules that can reach the surface, bind strongly, and reduce defects, both good stability and strong luminescence may be achieved.^24,28^ Likewise, the advantage of 2MPA over 3MPA in enhancing the luminescence intensity and colloidal stability due to the stability of 2MPA and energetically favorable bidentate surface binding compared to 3MPA has been demonstrated on CdS QDs, and this approach was successfully utilized in Ag_2_S QDs by our group, as well.^29^ We suggest that these coating molecules and the stabilization strategy could also provide small, stable, strongly luminescent AIS QDs without the Zn dopant or ZnS shell and produce an AIS portfolio with opposite surface charges and emission in the medical imaging window.

Achievement of such aqueous cationic and anionic AIS QDs with the emission in the visible region that can be prepared with either 2MPA or 2MPA/PEI in surface charge would facilitate their evaluation in nanomedicine. Currently, evaluation of AIS or ZAIS QDs in medicine is quite limited and mostly focuses on toxicity evaluation and in vitro.^10,11,21^ Here, these AIS QDs are also evaluated as a theranostic agent for photodynamic therapy (PDT).

PDT is a light-based medical treatment that relies on the retention of photosensitizers (PSs) in tumor tissues, which can be activated at a specific wavelength to provide a therapeutic effect at the irradiated area. PSs activated by a specific wavelength in the presence of molecular oxygen generate singlet oxygen and other reactive oxygen species (ROS), which are cytotoxic to the nearby cells due to the rapid and dramatic increase in oxidative stress.^30−32^ Hence, PDT is quite effective in eliminating various pathogens such as viruses and bacteria as well as cancer cells and therefore has found use in a variety of areas including dermatology, ophthalmology dentistry, cosmetics, treatment of some cancers, microbial/viral infections/biofilms, and sterilization. In addition, photochemical internalization (PCI) of toxic drugs has become an attractive strategy.^33−39^

5-Aminolevulinic acid (ALA) is a prodrug and the precursor of protoporphyrin IX (PpIX), a natural PS and an intermediate in the heme biosynthesis pathway.^40−42^ ALA is clinically approved for PDT and fluorescence-based photodiagnosis of different cancers due to the efficient metabolism of the fluorescent PpIX photosensitizer in the malignant tumor cells. In clinical practice, red wavelengths are typically employed for ALA-PDT, whereas fluorescence photodiagnostic imaging typically uses blue light excitation. However, ALA has relatively low bioavailability and cellular uptake owing to its zwitterionic nature. Hence, its derivatization and loading to nanoparticles have been proposed for better therapeutic outcomes. Preparation of ALA derivatives with improved bioavailability and lipophilicity via esterification, their peptide esters, and dendritic and liposomal formulations have been investigated.^43−45^ Chung et al. increased the cellular uptake of ALA by conjugating it to methoxy polyethylene glycol/chitosan.^46^ Indeed, the utilization of nanoparticles in ALA-based PDT improves the stability of PS and could also be tuned to deliver it to the target cells in high concentration to improve the therapeutic effect and evade the efflux transporters that mediate drug resistance.^47^ Wang et al.^48^ increased the circulation time and stability of ALA by loading it into HER2-targeted aldehyde-functionalized hyaluronic acid and hydroxyethyl chitosan nanoparticles. They showed that less than 40% of ALA was released at physiological pH after 45 h, whereas the half-life of free ALA under the same conditions is less than an hour.^48^

ALA may be conjugated to the nanoparticles, or it may be electrostatically loaded to the charged nanoparticles.^46,49^ One advantage of electrostatic loading is the fact that it can be done in fewer steps. Procedures involving multiple solvent exchanges, surface functionalization, washing, etc., usually negatively affect the luminescence intensity and hydrodynamic size of QDs. Another advantage of electrostatic loading is pH-dependent, quick release with higher efficiency, especially in acidic media, which is important for clinical applications. The few examples of electrostatic loading in the literature utilized the positively charged gold nanoparticles.^49,50^

We suggest that ALA can be loaded electrostatically to both the cationic and anionic nanoparticles due to its zwitterionic nature. Yet, there are no studies comparing the cationic versus anionic nanoparticles as the delivery vehicles for ALA. The charge of the nanoparticle would impact the biodistribution, dark toxicity, and probably the binding and release efficiency of ALA, which are critical in ALA delivery with nanoparticles for improved PDT efficacy. The current studies related to the relationship between the cellular uptake and physicochemical properties of the nanoparticles in the absence of targeting agents imply that the access of QDs into the cells is principally governed by the size and charge of the nanoparticles.^51,52^ The negatively charged proteoglycans are supposed to attract the positively charged QDs toward the cell membrane, causing an increased endocytic uptake of the cationic nanoparticles but repelling the negatively charged ones resulting in a lower uptake.^53^ However, this is still disputed because some papers report that the negatively charged nanoparticles are endocytosed more than the positively charged QDs, whereas other studies show no correlation of the QD surface charge.^54−56^ In the present study, using AIS with different coatings provides a great tool to answer these questions.

Herein, we developed both the cationic and anionic AIS QDs with strong luminescence in one-step aqueous synthesis without Zn incorporation or ZnO shell, simply by utilizing either PEI/2MPA or 2MPA as the stabilizers and loading them with ALA as the potential theranostic agents for tumor therapy. With this design, the QDs will, in principle, protect the ALA and enable its accumulation in the tumors due to the EPR effect and endocytic cell internalization and provide a strong and long-lived QD-based luminescence highlighting the region of accumulation and favor the ALA release in the tumors selectively, hence causing a significant increase in the tumor PpIX levels, which will lead to enhanced cell death when irradiated.^28^

**Scheme 1:**
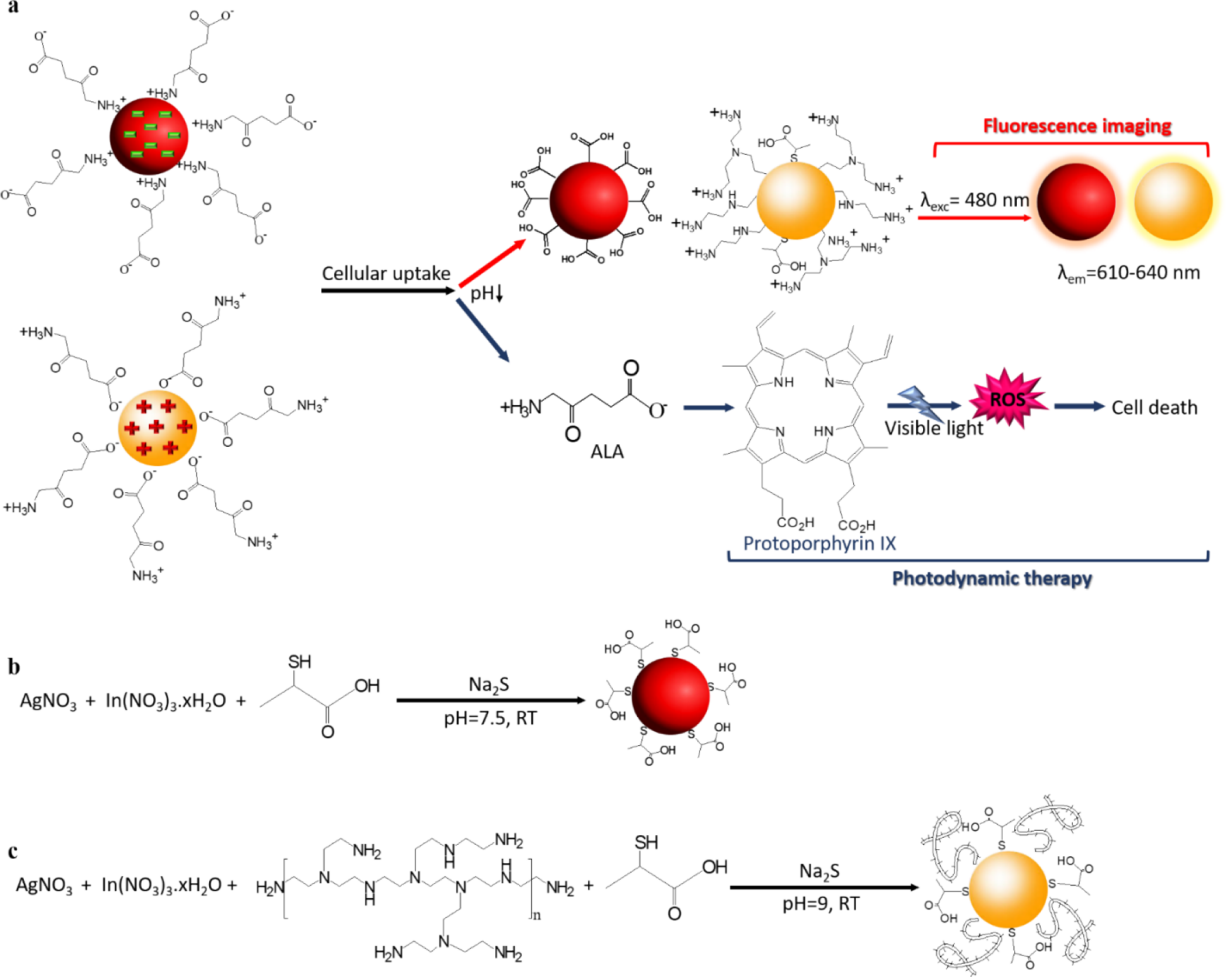
(a) Use of the ALA-Loaded Cationic and Anionic AIS QDs for Visible Light PDT Coupled with QD-Based Optical Imaging in the Medical Imaging Window. Synthesis of (b) AIS-2MPA and (c) AIS-PEI/2MPA QDs

The AIS QDs prepared from silver nitrate, indium(III) nitrate, and sodium sulfide in different Ag/In/S/coating ratios provide the stable cationic and anionic AIS QDs emitting from green to orange-red. Bandgap and Urbach energies were determined to correlate the size, size distribution, defects, and emission wavelength of the particles. Long-term emission stability, as well as the colloidal stability, was monitored. Then, in vitro fluorescence, cytotoxicity, and cell internalization of the cationic and anionic AIS QDs were examined in the 2D and 3D-spheroid models of the HT29, HeLa, SW480, and HCT116 cancer cell lines. Finally, the potential for ALA-based PDT was investigated, and the cationic and anionic AIS QDs as the delivery vehicles of ALA were compared. The ALA loading and pH-dependent release efficiency from both the QDs and PDT efficiency of the AIS/ALA QD compositions upon 5 min 420 nm (UV lamp) irradiation were examined (Scheme 1a).

Overall, the results demonstrate that the 2MPA and PEI/2MPA coatings do successfully stabilize the AIS QDs to provide small, stable QDs with strong emission; such QDs have strong luminescence in the optical imaging window, and ALA may be effectively loaded and released from both, and the QD-delivered ALA provides greater phototoxicity to the cancer cell lines. The promising results have opened possibilities for the bio-labeling and image-guided PDT of cancer utilizing these AIS/ALA QDs, which may be adopted to deliver other therapeutic cargos.

## Experimental Section

### Materials

Silver nitrate (AgNO_3_), In(NO_3_)_3_·*x*H_2_O, and branched polyethyleneimine (PEI) (Mw 25 kDa) were purchased from Sigma-Aldrich (Germany). Sodium sulfide (Na_2_S) was purchased from Alfa-Aesar. Sodium hydroxide (NaOH), 2-mercaptopropionic acid (2MPA), ethanol, and acetic acid (CH_3_COOH) were purchased from Merck (USA). 5-Aminolevulinic acid hydrochloride (ALA) was bought from Research Products International (RPI-USA). The LDS 798 near-IR laser dye was purchased from Exciton, Inc., (USA). Ultracentrifuge tubes with polysulfone filtration membranes (3 kDa) were purchased from Sartorius (Germany). All chemicals were of analytical grade and used without any further purification.

The Roswell Park Memorial Institute (RPMI) 1640 and McCoy’s 5A (modified) medium were obtained from Multicell, Wisent Inc. (Canada). The 10× minimum essential medium (MEM) (used as the color/pH indicator) and HEPES buffer solution were purchased from Gibco by Life Technologies, Thermo Fisher Scientific (UK). Fetal bovine serum and l-glutamine were purchased from Capricorn Scientific GmbH (Germany). 80% Rat Tail Collagen Type I was bought from First Link UK Ltd. Trypsin–EDTA and penicillin–streptomycin solutions were bought from Wisent Inc. (Canada). Thiazolyl blue tetrazolium bromide (MTT), Alamar blue, and phosphate buffered saline (PBS) tablets were purchased from Biomatik Corp. (Canada). Paraformaldehyde solution 4% in PBS was obtained from Santa Cruz Biotechnology, Inc. (USA). 96-well plates were purchased from Nest Biotechnology Co. Ltd. (China). The human epithelial carcinoma cell line (HeLa), HT29, and SW480 cells were provided by Gozuacik Lab (Sabanci University, Istanbul, Turkey) for this study. HCT116 cells were purchased from ATCC (Ireland).

### Synthesis of AIS-2MPA QDs

The 2MPA-coated AIS QDs were prepared at different Ag/In and Ag/S ratios as summarized in Table 1, keeping the 2MPA/cation mole ratio fixed at 5. All the reactions were performed under argon and at room temperature (RT). Briefly, 2MPA in the desired amounts based on the stoichiometries listed in Table 1 (2MPA/S: 1/0.25–1/3.3 molar ratio) was dissolved in 75 mL deoxygenated water in a round bottom flask. The pH of this solution was adjusted to 7.5 using NaOH and CH_3_COOH solutions (1 M), and then AgNO_3_ and In(NO_3_)_3_·*x*H_2_O were added. As an example, 0.022 mmol AgNO_3_, 0.22 mmol In(NO_3_)_3_·*x*H_2_O, and 1.2 mmol 2MPA were utilized for a molar ratio of Ag/In 1/10. The pH of the final solution was adjusted to 7.5 and then charged with a 25 mL aqueous solution of Na_2_S (0.22 mmol Na_2_S for a molar ratio of Ag/In/S 1/10/10) under vigorous mechanical stirring at 500 rpm. The samples were removed from the reaction mixture at different time points to track the particle growth. The prepared quantum dot solutions were washed with deionized water using the centrifugal filters (3 kDa cut off) and stored in the dark at 4 °C (Scheme 1b).

**Table 1:** Effects of the Different Reaction Variables on the Properties of the AIS-2MPA QDs

sample	AIS-2MPA-1	AIS-2MPA-2	AIS-2MPA-3	AIS-2MPA-4	AIS-2MPA-5
Ag/S ratio	1/10	1/2.5	1/10	1/1.25	1/10
Ag/In ratio	1/4	1/4	1/10	1/4	1/2
cation/S ratio	0.5	2	1.1	4	0.3
λ_emi_^a^ (nm)	634	660	630	701	649
bandgap (eV)	2.10	2.13	2.15	2.34	2.15
*E*_u_^b^ (meV)	172	168	170	137	161
FWHM^c^ (nm)	161	190	80	203	219
Dh-number^d^ (nm)	46.1 ± 1.6		5.2 ± 1.1	103.7 ± 2.4	22.6 ± 2.5
Dh-intensity^e^ (nm)	80.7 ± 10.3		38.2 ± 11.2	117.9 ± 20.1	67.9 ± 5.7
zeta potential (mv)	–38.1 ± 5.2		–42.1 ± 1.9	–43.8 ± 3.1	–41.6 ± 4.4
QY (%)	10.6 ± 2.1		19.4 ± 2.7	5.6 ± 2.1	4.9 ± 0.9

a Emission maxima.

b Urbach energy.

c Full-width at half-maximum of the emission peak.

d Hydrodynamic diameter measured by DLS and reported as the number average.

e Hydrodynamic diameter measured by DLS and reported as the intensity average.

### Synthesis of AIS-PEI/2MPA QDs

The PEI/2MPA-coated AIS QDs were prepared at different Ag/In and Ag/S ratios as summarized in Table 2 at a coating/cation mole ratio of 5. All the reactions were performed under argon and at RT. The 4/1 mole ratio of PEI/2MPA was used in all the reactions based on the amine/thiol content of the species. The cation sources (AgNO_3_, In(NO_3_)_3_·*x*H_2_O) and Na_2_S were dissolved in 75 and 25 mL of deoxygenated water, respectively, in separate round bottom flasks. In a typical reaction for a molar ratio of Ag/In/S 1/10/10, 0.2 mmol PEI and 0.05 mmol 2MPA were added to the 0.022 mmol AgNO_3_ and 0.22 mmol In(NO_3_)_3_·*x*H_2_O solution. The pH of the final solution was adjusted to 9 using NaOH and CH_3_COOH solutions (1 M). Na_2_S solution (0.22 mmol Na_2_S for a molar ratio of Ag/In/S 1/10/10) was added to this reaction mixture under vigorous mechanical stirring at 500 rpm. The samples were removed during the reaction at different time points to follow the particle growth. In the end, the quantum dot solutions were washed and kept as described above (Scheme 1c).

**Table 2:** Effect of the Different Reaction Variables on the Properties of the AIS-PEI/2MPA QDs

sample	AIS-PEI/2MPA-1	AIS-PEI/2MPA-2	AIS-PEI/2MPA-3	AIS-PEI/2MPA-4	AIS-PEI/2MPA-5	AIS-PEI/2MPA-6	AIS-PEI/2MPA-7
Ag/S ratio	1/10	1/10	1/2.5	1/10	1/5.5	1/1.25	1/10
Ag/In ratio	1/10	1/4	1/4	1/0.25	1/10	1/4	1/2
cations/S ratio	1.1	0.5	2	0.5	2	4	0.3
λ_emi_^a^ (nm)	617	640	618	774	618	655	638
bandgap (eV)	2.74	2.4	2.57	2.02	2.15	2.12	2.24
*E*_u_^b^ (meV)	138	149	144	167	168	151	150
FWHM^c^ (nm)	147	177	147	105	139	240	189
Dh-number^d^ (nm)	7.7 ± 0.9	12.7 ± 0.4	10.5 ± 0.6	11.9 ± 0.4		8.2 ± 1.3	57.9 ± 3.7
Dh-intensity^e^ (nm)	27.9 ± 4.1	26.3 ± 2.2	37.9 ± 10.3	60.4 ± 6.8		17.6 ± 8.7	99.1 ± 10.3
zeta potential (mV)	10.7 ± 2.1	8.1 ± 1	9.8 ± 0.3	16.4 ± 0.9		20.9 ± 3.2	–4.9 ± 1.7
QY (%)	20.3 ± 1.4	15.6 ± 0.9	7.9 ± 0.4				

a Emission maxima.

b Urbach energy.

c Full-width at half-maximum of the emission peak.

d Hydrodynamic diameter measured by DLS and reported as the number average.

e Hydrodynamic diameter measured by DLS and reported as the intensity average.

### Characterization

X-ray photoelectron spectroscopy (XPS) using Thermo Scientific K-Alpha XPS with Al K-alpha monochromatic radiation (1486.3 eV) was used for compositional analysis of the crystals. The dried samples were placed on an adhesive carbon tape and exposed to 400 μm X-ray spot size with 50.0 eV pass energy, corresponding to a resolution of roughly 0.5 eV. All the spectra were corrected with respect to the C1s peak at 284.5 eV. To determine the crystal structure of the AIS QDs, a Bruker D2 Phaser Benchtop X-ray Diffraction (XRD) system with Cu K-α radiation (λ = 1.5406 Å) was used between 2θ angles of 10 and 80°. The hydrodynamic sizes, zeta potentials, and polydispersity index (PDI) of the colloidal AIS QDs were assessed with a Malvern zetasizer nano ZS. The Ag and In contents of the colloidal AIS QDs were determined quantitatively by Agilent 7700x Inductively Coupled Plasma Mass Spectrometry (ICP-MS) after the samples were treated with nitric acid:sulfuric acid (9:1 v/v). This was performed after sterile filtration of the colloidal solutions to have the exact concentrations used in the in vitro studies. The functional group analysis of the QDs was performed on a Thermo Scientific Nicolet iS10 instrument (FTIR) in the wavenumber region from 650 to 4000 cm^–1^ with resolution 4 cm^–1^. The organic content of the QDs was measured by thermogravimetric analysis (TGA, TGA 500). The samples were heated from RT to 1000 °C at a heating rate of 10 °C/min under a nitrogen atmosphere.

The photoluminescence (PL) spectra of the AIS QDs in the range of 500–900 nm were recorded on an Agilent Cary Eclipse Fluorescence Spectrophotometer at 480 nm excitation. The PL lifetimes were measured using an Edinburgh FLS100 Spectrometer. The absorbance spectra were recorded using a Shimadzu UV-3600 spectrophotometer in the 300–1000 nm range. The QY was calculated based on the procedures in the literature using Rhodamine 6G as reference according to the following equation:^24^
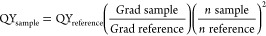
*G*rad is the ratio of the fluorescence intensity to the absorbance of the corresponding sample, and *n* is the refractive index of the solvent.

### Cell Culture

All culture media were supplemented with 10% fetal bovine serum, 2% l-glutamine (200 mM), and 1% penicillin–streptomycin. HT29, SW480, and HCT116 were cultured in McCoy’s, and HeLa cells were cultured in the RPMI 1640 complete medium in a 5% CO_2_-humidified incubator at 37 °C.

### Formation of 3D Spheroid Constructs

The in vitro 3D-spheroid constructs were prepared following the protocol from RAFT 3D culture systems (Lonza, Slough, UK). The hydrogels were prepared from a mixture containing 10% 10× MEM (used as the color/pH indicator) and 80% Rat Tail Collagen Type I (First Link UK Ltd. Custom Bio-Reagents) and neutralized with a solution made from 1.65 M NaOH and 840 mM HEPES buffer (Thermo Fisher Scientific). The cells were seeded into the collagen at densities of 50,000 cells for the spheroid constructs at overall (cells and collagen mix) volumes of 240 μL per well in a 96-well plate. The constructs were incubated at 37 °C for 15 min to set before being subjected to plastic compression using absorbers (Lonza) for a further 15 min at RT. After the removal of the absorbers, fresh medium was added, and the wells were placed into the incubator.

### Cytotoxicity Assay

The MTT metabolic activity assay was used to determine the dark cytotoxicity of the AIS-2MPA and AIS-PEI/2MPA QDs. All the QD colloidal solutions were sterilized by passing through sterile 0.2 μm filters. HT29, HeLa, SW480, and HCT116 cells were seeded into a 96-well plate at a density of 12,500 cells/well and cultured at 37 °C in a 5% CO_2_ atmosphere. The medium was replaced with a fresh medium after an overnight exposure with the QDs in cation concentrations of 0.025–100 μg/mL as determined by ICP analysis. After 48 h incubation, the medium was replenished, and the cells were washed with 3× PBS. After the addition of 50 μL of MTT reagent (5 mg/mL) and 150 μL medium to each well, purple formazan crystals formed in 4 h. The cells were washed with 3× PBS, and then the crystals in each well were dissolved with ethanol:DMSO (1:1 v/v) solution by shaking gently for 15 min. Relative cell viability was determined based on the absorbance intensity measured at 600 nm with a reference reading at 630 nm using a microplate reader (BioTek ELx800 Absorbance Microplate Reader). Cells with no treatment were used as controls. Treated cells without the MTT reagent were used for the correction of the absorbance intensities.

### Cell Uptake and Imaging

The first experiments were performed on the 2D cell culture and then on the 3D spheroids consisting of multicellular aggregates that grew over 7 days before treatment. HT29, HeLa, SW480, and HCT116 cells were seeded on 3 mL glass-bottom petri dishes at a density of 1.75 × 10^5^ cells/well in a complete medium and grown for 24 h at 37 °C and 5% CO_2_. On the next day, the growing cells were incubated with nanoparticles at 50 and 2 μg/mL cation concentrations of AIS-2MPA-3 and AIS-PEI/2MPA-1 QDs for 24 h. Then, the medium was replenished, and the cells were washed with 3× PBS and were fixed with paraformaldehyde solution (4% in PBS) for 20 min. After being washed with PBS, the cells were incubated for an additional 15 min with 2 μg/mL of DAPI nucleus dye. The cells were rewashed three times with PBS to remove the unbound dye and left in 2 mL PBS to prevent drying. Cells without any QD treatment were used as controls. The fixed cell samples were examined using a confocal microscope (Leica dmi8/SP8) with different filters for Alexa 488 (λ_exc_: 488 nm and λ_em_: 580–780) and DAPI (λ_exc_: 325–375 nm and λ_em_: 435–485 nm). The same experimental procedure was also performed for the control cells that were not treated with the nanoparticles. Images were processed and merged using the ImageJ analysis program.

The quantitative analysis of QD uptake was performed by the determination of cation (Ag and In) content in the QD-treated cells. HT29, HeLa, SW480, and HCT116 cells were prepared similarly as for microscopy image analysis and incubated with the QD samples for 24 h. Then, the cells were washed with PBS to remove the un-internalized nanoparticles, and after trypsinization, the supernatant was collected. An acid digestion procedure (suprapur nitric acid 65% and suprapur sulfuric acid 96%) was performed, and the cation concentration of each sample was determined by ICP-MS (*n* = 3).

To determine the intracellular localization of the QDs within the 3D constructs, separate constructs of HT29 and SW480 cell lines were prepared (50,000 cells/model). The constructs were incubated with AIS-2MPA-3 (50 and 2 μg/mL [cations]) and AIS-PEI/2MPA-1 (2 μg/mL [cations]) and their ALA conjugates for 24 h. The constructs were washed with PBS three times and incubated with PBS for fluorescence imaging using an Olympus fluorescence microscope (10× objective, Olympus BX63) with the following filters: BF (λ_exc_: 345 nm and λ_em_: 455) and TRITC (λ_exc_: 545 nm and λ_em_: 620 nm). Samples without QD treatment served as a control.

### Electrostatic Loading of ALA to AIS QDs

ALA at a concentration of 3 mg/mL was dissolved in 20 mM HEPES buffer, and its pH was adjusted to 7.2–7.4 and finally filtered from a 0.2 μm filter. The AIS-2MPA-3 and AIS-PEI/2MPA-1 QDs were also, separately, filtered from a 0.2 μm filter. Subsequently, the 0.3 and 0.5 mmol ALA solutions were mixed with 1 mmol AIS-2MPA-3 and AIS-PEI/2MPA-1 suspensions and stirred at RT for 15 min. ALA amounts were calculated as 30 and 50 mol % of the functional groups available on the QDs, which was determined by TGA. After loading, the nanoparticles were stored at 4 °C.

### Isothermal Titration Calorimetry (ITC)

An isothermal titration calorimeter (Affinity ITC, USA) was used to measure the enthalpy changes resulting from the interaction between ALA and either AIS-2MPA or AIS-PEI/2MPA QDs. One milliliter aliquots of either QD or ALA solution (1 mg/mL, pH 7.4) were injected sequentially into a 1.5 mL titration cell every 10 s. The temperature of the solution in the titration cell was maintained at 25 °C throughout the experiments. The results are reported as the change in enthalpy per titration (μcal) versus the concentration of the added ALA after each injection.

### ALA Release Profile

The release of the loaded ALA from the AIS QDs was investigated at pH 5.5 and 7.4 at 37 °C. Typically, 5 mL AIS-2MPA-3-50%ALA or AIS-PEI/2MPA-1-50%ALA was added to a dialysis bag (MWCO 3500) and immersed in 500 mL of PBS (pH 5.5 or 7.4) with constant shaking (100 rpm) at 37 °C. At fixed time intervals, 3 mL of the released solution was removed, and 3 mL fresh PBS medium was added. The amount of ALA in the removed samples was determined by absorbance at 263 nm and using a concentration-dependent absorbance standard curve prepared for ALA in PBS (pH 5 or 7.4).

### Measurement of PpIX Formation

HT29, HeLa, SW480, and HCT116 cells were seeded at a density of 12,500 cells/well in the 96-well plates and incubated for 24 h in a complete medium. The medium of some wells containing the cells was then replaced with a serum-free medium containing 0.002–0.688 mM ALA to be used as control, whereas the other wells were subjected to AIS-2MPA-3 and AIS-PEI/2MPA-1 containing the corresponding amount of ALA. Untreated cells were used as the drug-free control. The fluorescence intensity was measured using a Synergy H1 microplate reader (Biotek) at an excitation and an emission wavelength of 420 and 635 nm, respectively, after 24 h incubation with ALA or nanoparticles.

### PDT Studies

For the in vitro PDT studies, HT29 and SW480 cells were seeded at a density of 12,500 cells/well (2D cell culture) and 50,000 cells/well (3D models) in 96-well plates and cultured as described above for 24 h (the 2D cell cultures) and 7 days (3D models). The QDs containing different concentrations of ALA in the range of 0.018–0.688 mM were added to each well and incubated for 24 h. Then, the medium was substituted with a serum-free medium, and each well was illuminated with a blue LumiSource-flatbed lamp with a peak emission at 420 nm and 7 mW/cm^2^ output (PCI Biotech, Oslo, Norway) from the bottom of the plate for 5 min. The light-irradiated cells were then incubated for another 24 h before cell viability was determined using the standard MTT protocol for the 2D cell cultures and the fluorescence-based Alamar blue assay for the 3D models, which is the appropriate assay to use for light scattering constructs. The viability of cells that were treated the same way except laser treatment was also determined. Cells that were not treated with no ALA or QD treatment were used as controls.

### Intracellular ROS Generation

To assess ROS generation as a result of irradiation, HT29 and SW480 cells were seeded in the 96-well plate at a density of 12,500 cells/well (2D cell cultures) and incubated in complete media for 24 h. Then, QDs were added to the cells at cation concentrations of 50 and 2 μg/mL of AIS-2MPA-3 and AIS-PEI/2MPA-1 QDs for 24 h, respectively. The cells were then also irradiated using a blue LumiSource-flatbed lamp with peak emission at 420 nm and 7 mW/cm^2^ output (PCI Biotech, Oslo, Norway) for 5 min and incubated for another 24 h. The ROS levels of the treated cells were determined using the Enzo Total ROS Detection Kit following the manufacturer’s instructions using a Synergy H1 microplate reader (Biotek) at an excitation/emission wavelength of 485/538 nm.

### Live/Dead Assay

A live/dead cell viability assay (Thermo Fisher Scientific, UK) was utilized to determine the live and dead cell population after the ALA, QD, and ALA-loaded QD treatment before and after irradiation. HT29 and SW480 cells were seeded following the procedure explained for the ROS assay. After irradiation at 420 nm for 5 min, the cells were stained with the live/dead assay based on the protocol that is provided by the manufacturer. Finally, images were collected using an inverted fluorescence microscope (EVOS FL color, Life Technologies, Thermo Fisher Scientific, UK).

### Termination of Apoptosis/Necrosis

The Annexin V-FITC apoptosis detection kit (Abcam, UK) was used to confirm the mechanism of cell death in the 2D and 3D models. The cells were seeded as described above. At 24 h after being exposed to irradiation at 420 nm, 100 μL of 1× binding buffer was added to the models in each well before adding 1 μL of Annexin V-FITC (Ex/Em 488/525 nm) and 5 μL of propidium iodide (Ex/Em 535/617 nm) to the buffer solution in each well. The stained cells were then imaged using an EVOS fluorescence microscope (EVOS FL color, Life Technologies, Thermo Fisher Scientific, UK).

### Statistical Analysis

Two-way ANOVA with Tukey’s multiple comparison test was used for statistical analysis from the GraphPad Prism 8 software package from GraphPad Software, Inc., USA. All data were expressed as mean ± standard deviation (SD), and *p* < 0.05 was considered statistically significant.

## Results and Discussion

### Synthesis and Characterization of AIS-2MPA QDs

**Figure 1:**
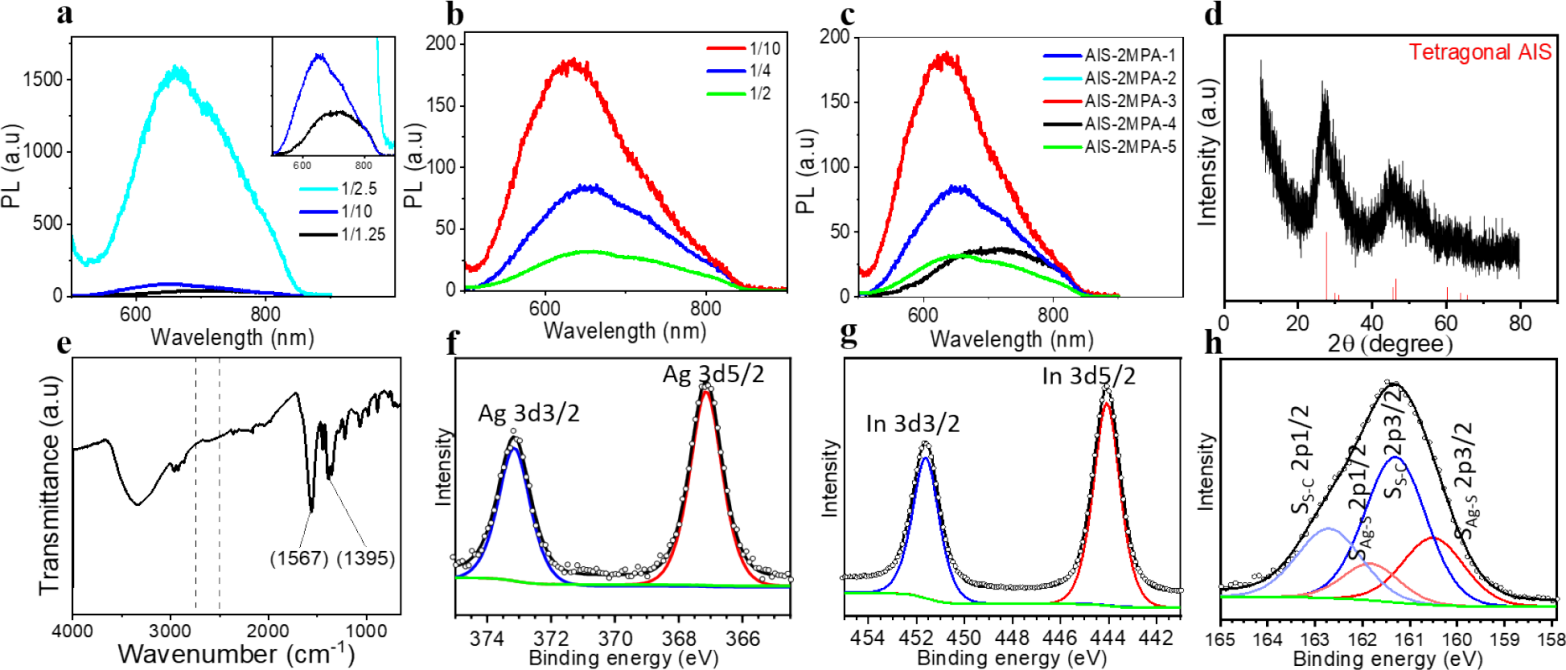
(a) PL spectra of the AIS-2MPA QDs prepared with a constant molar ratio of Ag/In 1/4, cations/coating 1/5, and different molar ratios of Ag/S. (b) PL spectra of the AIS-2MPA QDs prepared with a constant molar ratio of Ag/S 1/10, cations/coating 1/5, and different molar ratios of Ag/In. (c) PL spectra of all the AIS-2MPA QDs prepared at different conditions. (d) XRD (AIS JCPDS: 25-1328), (e) FTIR, (f) Ag 3d, (g) In 3d, and (h) S 2p XPS spectra of the AIS-2MPA-3 QDs.

One of the major hurdles in nanotechnology is the stability of nanoparticles.^58^ 2MPA was shown to be a good coating material because it dramatically increases the stability of the nanoparticles and enhances the luminescence intensity.^29,59^ Hence, anionic AgInS_2_ (AIS) QDs were synthesized under aqueous conditions in the presence of 2MPA at RT at different Ag/In/S/2MPA ratios, and their influence on the optical properties and stability of QDs was determined (Table 1, Figure 1, Figure S1). All the emission peaks were quite broad, with FWHM between 79 and 219 nm (Table 1). Such a broad emission profile (Figure 1) coupled with the featureless absorbance spectrum (Figure S1) of such ternary QDs is attributed not only to size distribution but also to compositional inhomogeneity of the crystals, structural defects, and related mid-gap energy states, resulting in not only band-edge but also donor–acceptor recombination and trap state emissions.^7,19^ In general, these AIS-2MPA QDs show a major peak maximum around 630–660 nm and a shoulder at a longer wavelength of 705–715 nm. This is in line with the reported emission profiles for the AIS QDs and the explanation mentioned above about radiative events.^7,19,60^ Bandgap energy determination of AIS is relatively tricky due to the uncertainty in electron-hole mass values and long absorbance tail.^61^ The bandgaps of these QDs calculated from the absorption spectra by replotting the data in the coordinates of the Tauc equation were found as 2.10–2.34 eV (Figure S2, Table 1). These values agree well with the *E*_g_ reported for AIS.^62,63^ On the other hand, when these values are evaluated, an inevitable error originating from the long absorbance tails masking the exact position of the band edge should be taken into account.^62,64^ Such long absorbance tails are attributed to the localized defect states within the crystals providing the sub-band states and hence the long absorbance tails and broad emissions.^64^ The absorption tail is called the Urbach tail and is associated with the Urbach energy (*E*_u_) that can be calculated using the following:
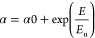
where α is the absorption coefficient, *E* is the photon energy (*h*ν), and *E*_u_ is the Urbach energy. By plotting the ln(α) against *E*, the Urbach energy can be calculated from the slope of the linear fit (Figure S3a–e).^63^ The Urbach energies of these AIS QDs are between 137 and 172 meV and show an increasing trend with the decreasing emission maxima, suggesting more disorder in the crystals as the size decreases. These *E*_g_ and Urbach energies agree with the values reported in the literature for the different anionic AIS QDs.^22,64^

### Influence of the Experimental Variables on the Optical Properties of AIS-2MPA QDs

#### Influence of the Ag/S Molar Ratio

To evaluate the effect of the Ag/S ratio (1/1.25, 1/2.5, and 1/10) on the optical properties of the produced QDs, varying amounts of the sulfur precursor were used while the other variables were fixed (Ag/In: 1/4, cations/coating: 1/5) (Table 1). Increasing the sulfur precursor blue-shifted the major peak maxima from 701 to 634 nm with an accompanying blue shift in the absorbance spectra (Figure 1a, Figure S4a).

These three recipes have indeed cation/S ratios of 0.5, 2, and 4 for AIS-2MPA-1, AIS-2MPA-2, and AIS-2MPA-4, respectively. So, in a way, as the Ag/S ratio decreased, the cation/S ratio also decreased, and a resultant blue shift in emission maxima was observed. This was probably due to an increasing stoichiometric imbalance as the formulations switched from cation-rich to S-rich, thereby reducing the crystal size. The smaller crystals were also accompanied by a stronger emission and a higher QY, which may be partially due to better passivation of the smaller crystals, lower degree of aggregation, and fewer crystal defects.

These observations are in line with the GSH-coated ZAIS reported by Deng et al.^7^ and the PEI-coated AIS reported by Raevskaya et al.^25^*E*_g_ decreased and *E*_u_ increased with the decreasing Ag/S ratio. The relatively large *E*_u_s and broad long-wavelength emission tail may support the significant emissions from the trap states. Here, a Ag/S ratio of 1/2.5 yielded the highest PL intensity. However, due to the lack of colloidal stability during the washing step, they could not be studied further. Because the QDs produced at a Ag/S molar ratio of 1/10 were the most luminescent and the stable QDs, this ratio was kept fixed to study the influence of the In/S ratio on the QD properties.

#### Influence of the Ag/In Molar Ratio

To further investigate the opportunity to tune the optical properties of the AIS-2MPA QDs, the ratio of Ag/S was fixed to 1/10, whereas the Ag/In ratio was varied as 1/2, 1/4, and 1/10 (molar ratio of cations/coating: 1/5) (Figure 1b, Figure S4b). Although the emission peaks were broad again, increasing the In content (decreasing Ag/In ratio) caused a slight blue shift in the emission maxima from 649 to 630 nm. This trend is in agreement with the earlier reports.^19,23^ Based on the HOMO–LUMO analysis of Tsuji et al., as the Ag content decreases, the hybrid orbitals of In5s and S3p become the major HOMO, instead of the hybrid orbitals of S 3p and Ag 4d, which increases the bandgap causing a blue shift in the emission.^65^

The Ag/In ratio substantially impacted the emission intensity, and the strongest emission was achieved at the highest Ag/In ratio of 1/10. The in-excess formulation is preferred due to the reactivity difference of Ag^+^ (soft acid) and In^3+^ (hard acid) with sulfide ( soft base) in the formation of ternary structures.^66,67^ Besides, it was suggested that higher QYs of In-rich recipes are correlated with an increased order in the In sublattices and reduction of intrinsic defects.^68^

A Ag/In ratio of 10 also corresponded to the cations/S ratio of 1.1 (AIS-2MPA-3), whereas others had a ratio of 0.5 (AIS-2MPA-1) and 0.3 (AIS-2MPA-5). Thus, this set’s cation-rich formulation may provide a better surface binding of the 2MPA and eliminate the surface defects. Indeed, when the emission profiles of these three QDs are examined, relatively strong intensities of the long-wavelength emission peaks are noticed, whereas AIS-2MPA-3 has a strong emission peak at 630 nm with quite a weak contribution of the long-wavelength emission (Figure 1b). This may be due to the reduced defects and related emissions that appear as the long-wavelength peak in these ternary QDs.^19^

To follow the growth of the particles, aliquots were taken from the reaction mixture at discrete time points, and their optical properties were examined, as it is believed that crystal growth kinetics play an important role in tuning the particle size and emission.^69^ Prolonged reaction times could influence crystallinity, change the crystal composition, and promote particle growth via the Ostwald ripening process.^19,70^ However, at extended reaction times, oxidation of the ligands, agglomeration of the nanoparticles, and the formation of defects may decrease the luminescence intensity.^71^Figure S1 shows the time-dependent changes in the emission and absorbance profile of the AIS-2MPA QDs. Nearly no change in the absorption onset was observed with the increase of growth time, suggesting no dramatic change in the size. The reaction duration mainly influenced the emission intensity rather than the peak maxima, suggesting that particle surfaces were well passivated but possibly underwent surface reconstruction.

The photoluminescence QYs of the AIS-2MPA-1 and AIS-2MPA-3 QDs are about 10 and 19% (Table 1). Hence, the best recipe for the synthesis of the colloidally stable and highly luminescence anionic AIS-2MPA has a molar ratio of Ag/In/S 1/10/10. Therefore, a new batch of AIS-2MPA-3 was synthesized and used in all the in vitro studies with emission maxima at 634 nm, 5.2 nm (number-based hydrodynamic size) diameter, and −37.5 mV zeta potential (Table S1).

### Structural Characterization of AIS-2MPA QDs

Structural characterization of AIS-2MPA was performed using the AIS-2MPA-3 QDs. The broad diffraction peaks detected via XRD analysis are attributed to tetragonal AIS QDs (Figure 1d).^19^ The FTIR spectrum of this QD provided information about the coating molecule (Figure 1e). The disappearance of the S–H stretching band, which would be between 2500 and 2540 cm^–1^, indicates the binding of 2MPA to the AIS core by its thiol.^10,59^ The stretching band of the C=O observed at 1567 cm^–1^ is consistent with the carboxylic acid interacting with the crystal surface. The broad absorption band at 3300 cm^–1^ can be attributed to the OH group, and the band at 1395 cm^–1^ corresponds to the C–O stretching vibrations of 2MPA bound to the AIS crystals, respectively.^10,72^ XPS analysis provided further information about the composition (Figure 1f–h). The Ag 3d core level peaks at binding energies (BE) of 367.10 eV (3d5/2) and 373.14 eV (3d3/2) fit well to the Ag^+^. The In 3d peaks at BE of 444.06 eV (3d5/2) and 451.58 eV (3d3/2) fit to the In^3+^ of AIS. The doublet at BE of 160.51 and 161.81 eV fits to the 2p3/2 and 2p1/2 of Ag–S, respectively. The other doublet at 161.30 and 162.71 eV belongs to the S of 2MPA.^7,57^ According to the XPS data, the Ag/In and Ag/S ratios were 1/7.5 and 1/11.5, which were consistent with the Ag/In ratio of 1/7 and Ag/S ratio of 1/12 determined by ICP. TGA analysis performed on the AIS-2MPA-3 QD indicates 62.8% organic content (Figure S4d). The TEM images of these QDs indicate small particles with an average diameter of 5.85 ± 0.74 nm (Figure S4e–f). EDS analysis shows colocalization of Ag and In and a broader distribution of S, which is in the core and the coating (Figure S4g).

### Synthesis and Characterization of AIS-PEI/2MPA QDs

**Figure 2:**
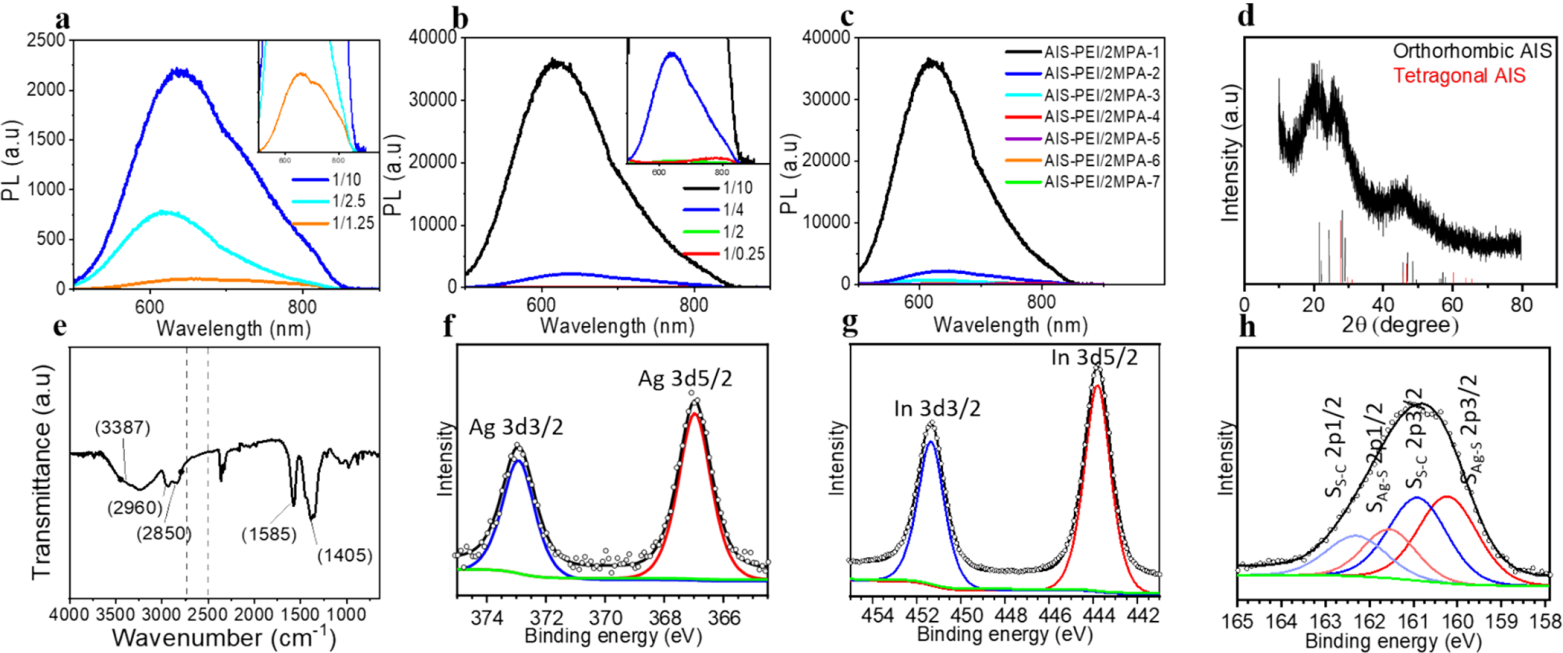
(a) PL spectra of the AIS-PEI/2MPA QDs prepared with a constant molar ratio of Ag/In 1/4, cations/coating 1/5, and different molar ratios of Ag/S. (b) PL spectra of the AIS-PEI/2MPA QDs prepared with a constant molar ratio of Ag/S 1/10, cations/coating 1/5, and different molar ratios of Ag/In. (c) PL spectra of all AIS-PEI/2MPA QDs prepared at different conditions. (d) XRD (AIS JCPDS: 25-1328), (e) FTIR, (f) Ag 3d, (g) In 3d, and (h) S 2p XPS spectra of the AIS-PEI/2MPA-1 QDs.

In developing the PEI-coated Ag_2_S, we have previously shown that PEI coating does not produce luminescent particles, but PEI/2MPA does.^73^ The most stable and luminescent Ag_2_S-PEI/2MPA QDs (AS-PEI/2MPA) were synthesized at pH 9 at a Ag/coating molar ratio of 1/5 and PEI/2MPA ratio of 4/1.^73^ Based on this knowledge, cationic AIS QDs were prepared with PEI/2MPA coating in water with slow addition of sulfide to the salt mixtures^7,9^ The introduction of Na_2_S into the solution containing Ag/In-PEI/2MPA produced a cloudy solution with yellow to brown coloration depending on the molar ratio of the components. The emission peak profiles of the AIS-PEI/2MPA QDs resemble those observed for AIS-2MPA: broad emission peaks with a long wavelength shoulder or tail due to various radiative coupling events involving the trap states and donor–acceptor pair recombination (Figure S6). With the different recipes wherein the stoichiometry of Ag/In/S was changed, AIS-PEI/2MPA QDs emitting in the red-NIR region with an emission peak maxima between 774 and 617 nm were obtained (Table 2, Figure 2, Figure S7a–g). The bandgap energies of these cationic AIS QDs were calculated between 2.02 and 2.74 eV from the Tauc plots (Figure S8) and Urbach energies between 138 and 168 meV (Figure S9). Indeed, these energies agree with the only reported PEI-coated AIS.^25^

### Influence of the Experimental Variables on the Optical Properties of AIS-2MPA QDs

#### Influence of the Ag/S Molar Ratio

The colloidal AIS-PEI/2MPA QDs synthesized with different Ag/S ratios at a constant Ag/In ratio of 1/4 and cations/coating ratio of 1/5 exhibited emission peaks with a maximum between 618 and 655 nm (AIS-PEI/2MPA-1, 3, and 6) with no significant tuning of the peak position. The dramatic difference in the PL intensity indicates stronger emission with the decreasing Ag/S ratio reaching its maximum at a Ag/S ratio of 1/10 (Figure 2a). Thus, this ratio is chosen as the optimum Ag/S ratio for the following experiments. Although there is no clear correlation between the PL peak maxima, bandgap energy, Urbach energy, and the Ag/S ratio (Figures S8, S9, and S10a), in general, bandgap widening with the decreasing Ag/S ratio was observed within this series (like the same trend in the AIS-2MPA QDs).^65,66^ These three recipes are all cation-rich, but excess Ag may be another source of defects increasing the *E*_u_.^25^

#### Influence of the Ag/In Molar Ratio

At a fixed Ag/S ratio of 1/10, the Ag/In ratio was varied from 1/0.25 to 1/10 (AIS-PEI/2MPA-1, 2, 4, and 7) (Table 2, Figure 2b) while keeping the other reaction conditions constant. A blue-shifted absorbance onset and emission peak maxima (from 774 to 617 nm) with the decreasing Ag/In molar ratio (increasing cation/S ratio) was observed as in AIS-2MPA (Figure S10b). The PL and UV–vis spectra of all QDs produced with the varied reaction variables are shown in Figure 2c and Figure S10c, respectively. According to Hu et al.,^71^ the blue-shifted emission with the decreasing content of Ag in the AIS QDs may originate from the defect-related recombinations instead of the transition between the quantized energy levels in the conduction and valence bands due to their large Stokes shift. Here, *E*_g_ increased from 2.02 to 2.7 eV with the decreasing Ag/In ratio, which supports blue-shifted spectra.^28,65^ The *E*_u_ increased from 138 to 167 meV with the increase in the Ag/In ratio (Table 2), indicating the increasing structural defects and disorders within the crystal (Figure S9).^58^ This indeed may indicate more crystal distortions and defects in the S-rich formulations. Overall, the most stable and luminescent cationic QDs are synthesized with a molar ratio of Ag/In/S 1/10/10 (AIS-PEI/2MPA-1) with ca. 20% QY, which is again a cation-rich formulation. These cationic QDs with emission maxima at 617 nm, zeta potential of 10.67 mV, and hydrodynamic size of 7.66/27.91 nm (number based/intensity-based average) were used for further characterizations and in vitro studies. This QD had the highest QY with a weak long wavelength tail in the emission spectra suggesting fewer defects in the crystals, which may be at least partially due to the cation-rich formulation that the ligands can better stabilize. Here, it is important to point out that the ultra-small cationic QDs (based on the hydrodynamic size) are obtained with such QY in a single step reaction at RT using the PEI/2MPA mixture. RT synthesis prevents S-release from mercaptoacids, and in the PEI coating, contribution of 2MPA provides strong surface binding and reduces the defects compared to the use of only PEI.

One example of cationic AIS QDs in the literature uses only PEI in reflux conditions producing 100 nm aggregates with 4–5 nm crystal size and achieved 20% QY (with respect to anthracene) only after 2 h of heat treatment at 100 °C.^25^ The other example to the pure PEI (10 kDa)-coated AIS QDs had a small hydrodynamic size and 32% QY but used a pressure cooker (120 °C) and did not report any long-term colloidal or luminescence stability.^22^ Both had maximum emission below 600 nm. These examples clearly indicate the advantage of using a mixed coating and its utility in tuning size emission and imparting strong emission in an RT reaction.

### Structural Characterization of AIS-PEI/2MPA QDs

The XRD pattern of AIS-PEI/2MPA-1 consists of the characteristic peaks of the tetragonal and orthorhombic AIS structure (Figure 2d).^74^ The FTIR spectrum of the AIS-PEI/2MPA-1 (Figure 2e) indicated the presence of the stretching vibrations at 1585 and 1405 cm^–1^, which are generally assigned to the binding mode of carboxylates to a surface or to a cation. The bands at 3387 and 2850–2960 cm^–1^ correspond to the stretching modes of the N–H and C–H bonds of PEI, respectively.^11^ The absence of the thiol peak in the range of 2500–2550 cm^–1^ confirms the covalent conjugation of the inorganic core to 2MPA via its thiol.^72^ XPS analysis provided further information about the AIS-PEI/2MPA-1 QDs. The XPS spectra obtained in Figure 2f–h confirmed the presence of the following elements: Ag 3d5/2 and 3d3/2 at BE of 372.92 and 336.94 eV and In 3d5/2 and 3d3/2 at BE of 451.38 and 443.79 eV. The two doublets were observed for S. BE of 160.22 (2p3/2)–162.57 eV (2p1/2) correspond to the core S, and 160.93 (2p3/2)–162.35 eV (2p1/2) corresponds to the bound S of 2MPA. These are consistent with the reported values for the AIS QDs and those observed for AIS-2MPA (Figure 2f–h).^74^ The N 1s region could be fitted to the two peaks at 398.20 and 401.12 eV, corresponding to the tertiary, secondary, and primary amines of PEI (Figure S10d). The high binding energy N 1s peak indicates primary amine binding to the crystal surface.^73^ The atomic ratio of the elements calculated from the area under each curve indicated a Ag/In/S ratio of 1/9/10, which is consistent with the theoretical ratio of 1/10/10. This ratio also agrees with the atomic ratio measured by ICP as Ag/In/S = 1/8.8/10.8. TGA analysis performed on the AIS-PEI/2MPA-1 QD indicates 58.6% organic content (Figure S10e). The TEM images of these QDs also indicate small particles with an average diameter of 6.1 ± 1.7 nm (Figure S10f–g). EDS analysis shows colocalized Ag and In with broader distribution of S, which is also coming from the 2MPA component of the mixed coating (Figure S10h).

### Colloidal Stability of the QDs

**Figure 3:**
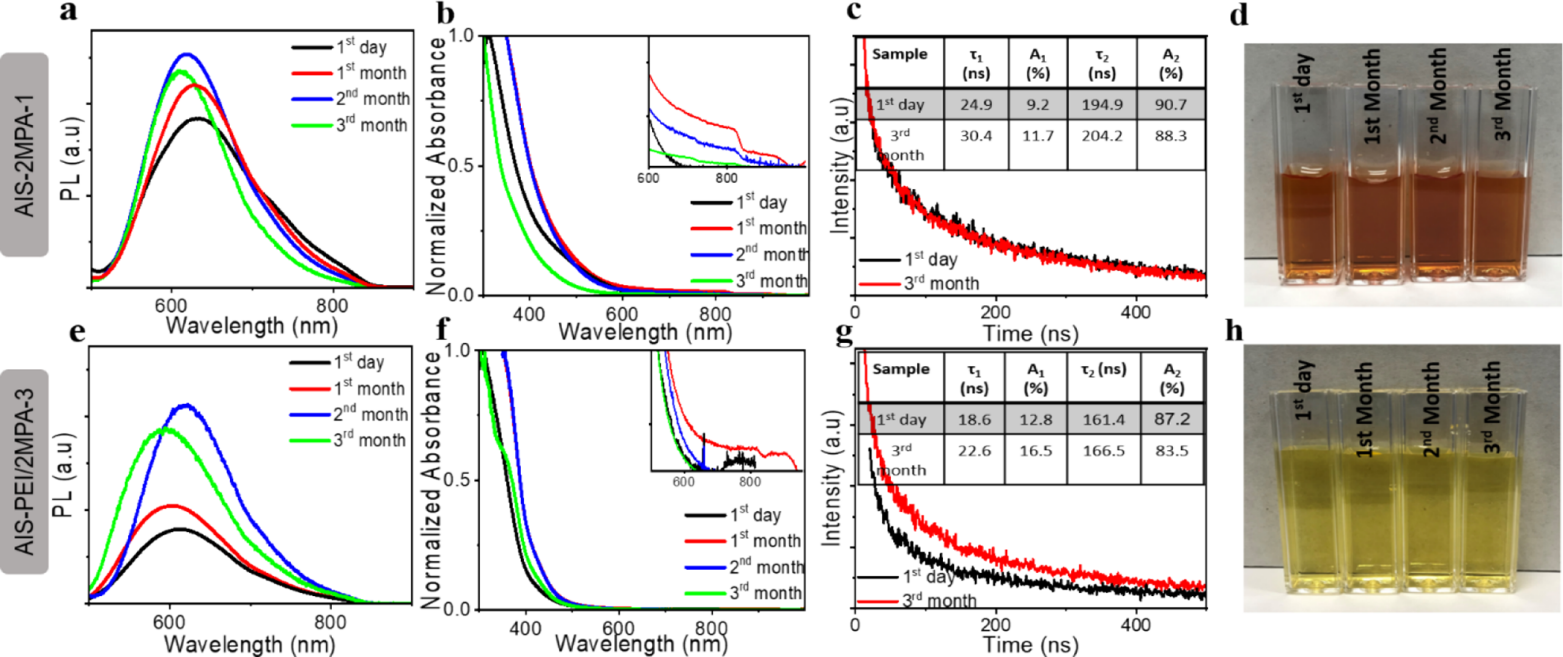
(a,e) PL spectra and (b,f) normalized UV–vis spectra and (c,g) PL lifetime of the AIS-2MPA-3 and AIS-PEI/2MPA-1 QDs at the 1st day and 3rd month of their synthesis, respectively. The inset shows the fit parameters of the decay curves. (d,h) Colloidal stability of the AIS-2MPA-3 and AIS-PEI/2MPA-1 QDs at different time points following its synthesis.

The shelf life of the nanoparticles is critical for any practical application. Generally, the PL intensity of QDs decreases over time due to the desorption of the ligands and/or oxidation, which may also cause an increase in the hydrodynamic size and precipitation.^73^ The emission and absorbance characteristics (PL and UV–vis spectra), hydrodynamic size, and zeta potential of the most luminescent QDs over a period of 3 months were tracked to determine the stability of the colloidal anionic and cationic AIS produced here (Figure 3 and Table S1). The colloidal QDs were stored at 4 °C after being washed. The emission intensity of both QDs increased with time, similar to Ag_2_S-2MPA, which was reported before (Figure 3a,e).^27^ To better understand the time dependence of PL intensity, the kinetics of the photogenerated charge carriers were studied using the time-resolved PL spectra of these QDs.

All the decay profiles were fitted according to the function of [*I*(*t*) = *A*_1_exp(−*t*/τ_1_) + *A*_2_exp(−*t*/τ_2_)], where τ_1_ and τ_2_ are the decay times and *A*_1_ and *A*_2_ are the fractional contributions of the two components.^6^ Generally, the fast component τ_1_ represents the bandgap recombination and the slow component, τ_2_ is related to the defect-related radiative recombination of the carriers.^7^ They were also suggested as high energy, intrinsic defect-related, donor–acceptor coupling versus low energy, surface effect-related emissions for the AIS QDs.^63^ For these AIS QDs, both τ_1_ (18–25 ns) and τ_2_ (161–165 ns) are relatively shorter than most AIS reported in the literature.^25,74^ The average lifetimes calculated from <τ> = (*A*_1_τ_1_^2^ + *A*_2_τ_2_^2^)/(*A*_1_τ_1_ + *A*_2_τ_2_) are also relatively shorter than the AIS QDs, which also have lower QYs than the ones produced here (inset table Figure 3c,g).^6^ The contribution of τ_1_, which was around 24.9 and 18.6 ns, was 9 and 13% for the AIS-2MPA-3 and AIS-PEI/2MPA-1 QDs, respectively. After 3 months of storage, τ_1_ and τ_2_ became longer, 30.4 and 22.6 ns, with an increased contribution, which is about 11.7 and 16.5% for the anionic and cationic AIS QDs, respectively. The ratio of *A*_2_/*A*_1_ decreased from 9.9 to 7.6 in AIS-2MPA-3 and from 6.8 to 5 in the AIS-PEI/2MPA-1 QDs after 3 months, indicating more contribution of the band edge and/or intrinsic defects than the surface defects with time.^23^ This was accompanied by a slight blue shift in the emission maxima from 632 to 612 nm for the anionic QDs and from 617 to 593 nm for the cationic AIS QDs (Table S1). The enhanced contribution of fast component, usually accompanied by a blue shift in the emission maxima of AIS or ZAIS QDs, indicates a lesser contribution of or say fewer surface defects.^25,60,63^ The hydrodynamic size of the QDs showed a slight increase after 3 months (Table S1). However, the high surface charge of both the AIS-2MPA-3 (−44.56 eV) and AIS-PEI/2MPA-1 QDs (11.75 eV) prevented any significant aggregation/precipitation, and they have maintained small hydrodynamic sizes that may be desired for any biological application (Table S1).

In summary, we can say that the particles are colloidally stable and no aggregation, precipitation, or quenching was observed after 3 months (Figure 3d,h), but instead, an enhancement in the luminescence intensity was detected (Figure 3a,e). Hence, they are very suitable for exploitation in in vitro studies.

### Electrostatic Loading of ALA to AIS-2MPA-3 and AIS-PEI/2MPA-1

ALA has one carboxylic acid and one primary amine group with p*K*_a_ values of 4.05 and 8.9, respectively. ALA should be zwitterionic at pH 7.2–7.4, and the electrostatic interaction between the −COO^–^ group of the AIS-2MPA-3 QDs and the −NH_3_^+^ group of ALA can allow the electrostatic loading of ALA to the anionic QDs. Two compositions were prepared with 30 and 50 mol % ALA loading (with respect to the 2MPA content of the QD) to AIS-2MPA-3. This increased the intensity-based hydrodynamic size of the AIS-2MPA-3 QDs from 38 to 62 and 50 nm, respectively, in HEPES buffer (pH = 7.2) (Table S2). This indicates some aggregation; however, the number-based size average does not indicate a dramatic aggregation (5.2 nm versus 5.7–5.9 nm). The zeta potential of the QDs changed slightly from −42 mV to −43 and −44 mV for 30 and 50% ALA loadings, respectively (Table S2). Although the carboxylate groups of the QDs are consumed by the NH_3_^+^ groups of ALA, the −COO^–^ groups of ALA reside on the periphery after binding; therefore, the zeta potential did not change much. In the AIS-PEI/2MPA-1 QDs, at pH 7.2 in HEPES buffer, the −NH_3_^+^ groups of QDs are electrostatically bound to the −COO^–^ groups of ALA. This increased the charge of the AIS-PEI/2MPA-1-30%ALA and AIS-PEI/2MPA-1-50%ALA QDs from ca. 11 to 12 and 16 mV, respectively (Table S2). This prevented aggregation, and the hydrodynamic size stayed around 27–33 nm with a reduced size distribution (Table S2). Again, the number-based average hydrodynamic size increased slightly from 7.6 to 9–10 nm with the ALA loading (Table S2). Overall, after the ALA loading, both QDs maintained small hydrodynamic sizes and their original charge character as desired for this study.

ITC has been used further to study the interaction between ALA and the QDs. Titration of QDs at pH 7.2 for AIS-2MAP-3 and AIS-PEI/2MAP-1 with 50 mol % ALA dissolved in HEPES buffer created an exotherm, which is an indication of binding (Figure S11a,b). The exotherm regularly decreased with the addition of ALA and reached almost to a constant value at the last injection. This suggests that ALA fully binds to both cationic and anionic AIS QDs at the studied doses. Yet, the binding exotherm is much stronger in the cationic AIS QDs, indicating stronger binding (Figure S11a,b).

### ALA Release Profile

The best drug delivery vehicles are supposed to bind strongly to the drug at the physiological pH and then release it effectively at the diseased site.^75^ Because ALA is loaded to the QDs via the electrostatic interaction, the pH changes should affect its release. AIS-2MPA-3-50%ALA and AIS-PEI/2MPA-1-50%ALA were incubated in PBS at acidic and neutral pH (5.5 and 7.4) for 40 h at 37 °C. Protonation of the carboxylic acids in acidic pH would weaken the electrostatic interaction between the anionic QD and ALA, causing its release. In the cationic QDs with the PEI content, protonation of amines and the 2MPA component will also weaken the interactions as ALA may have started to protonate as well. In the in vitro studies, the effective release of the anionic cargo from the PEI-containing delivery vehicles is also associated with the proton-sponge effect. Here, both QDs kept the release of ALA ≤15% at pH 7.4 in 24–40 h. A small burst release around 10% was followed by a sustained release profile in acidic media. In general, the release from the cationic QDs was slightly faster, reaching 30% in 5 h and a maximum value of 80% in 25 h. The ALA release from the anionic QD was about 20% in 5 h and 72% after 31 h in acidic pH. The enhanced ALA release in the acidic media should facilitate the therapeutic efficacy of the conjugates under the acidic tumor microenvironment in vivo. The emission intensity of the QDs after the 40 h ALA release at pH 5.5 showed only 20–30% reduction (Figure S11e–h), suggesting that appreciable stability of the QDs can be attained in a tumor microenvironment or acidic intracellular compartment. Xu et al. have demonstrated that ALA conjugated to gold nanorods via hydrazone linkage, which hydrolyzes under an acidic tumor environment, release 71% of ALA at pH 5 in 24 h and only 6% of ALA at neutral pH.^80^ In a study conducted by our group, the ALA release from the electrostatic-loaded ALA to anionic Ag_2_S QDs was reported ∼50% after 24 h in acidic media, whereas it was ∼25% at neutral pH.^76^ It is quite promising to see similar pH selectivity in release with the simply electrostatically loaded ALA to our QDs. The ALA release was much less in amount with poor pH dependence from the self-assembled polysaccharide-based nanocomplexes reported by Wang et al., which trapped ALA non-covalently. They reported 15 and 20% release of ALA in 5 h and 30% and 50% after 24 h at pH 7.4 and pH 5.5, respectively.^48^ Here, both the cationic and anionic QDs effectively protect ALA under physiological pH, which would minimize the leakage during circulation, but release the prodrug effectively at the endosomal/lysosomal pH, nominating these QDs as promising candidates for successful ALA delivery. The strong luminescence of the AIS QDs would also provide image-guided therapy of the are occupied by the nanoparticles and hence ALA, which would aid the operator to decide when to irradiate and highlight the sensitized region for the high level of locality in the treatment, which is undoubtedly very desirable for the improved therapeutic outcome with reduced side effects.

### Evaluation of In Vitro Dark Cytotoxicity of the QDs

Cytotoxicity of the AIS-2MPA-3 and AIS-PEI/2MPA-1 QDs and their ALA conjugates on various tumor cells was determined using the 2D cell culture of the HT29, HeLa, SW480, and HCT116 cell lines by the MTT assay (Figure S12). The QD doses were based on the total cation concentration of each QD determined by ICP-MS and studied in the range of 0.25–100 μg/mL. The AIS-2MPA-3 QDs did not show any significant cytotoxicity in this range after 48 h incubation (Figure S12a–d). The AIS-PEI/2MPA-1 QDs significantly impaired the cell viability of all tested cell lines at cation concentrations above 2 μg/mL (Figure S12e–h). PEI has a dose- and molecular weight-dependent toxicity due to its cationic nature, which is suppressed significantly by adsorption on the QD surface.^73^ Hence, the cationic nanoparticles are expected to be more toxic. Free ALA did not show any substantial cytotoxicity in either of the tested cell lines up to 0.69 mM, the highest concentration delivered at 100 μg/mL cation concentration. Although the cell viability slightly decreased when exposed to the ALA-loaded cationic QDs compared to the free QDs, ALA association with QDs did not lead to a dramatic difference between different formulations. The influence of these QDs on the viability of healthy colorectal cells was also tested. There was no loss in the viability of the CCD841 cells that were exposed to free ALA, AIS-2MPA-3 QDs (up to 200 μg/mL [cations]), or ALA-loaded AIS-2MPA-3 QDs (Figure S13a). In the AIS-PEI/2MPA-1 QDs and its ALA-loaded formulations, a significant drop in the cell viability was observed at 10 μg/mL cation concentration, which is mostly due to the cationic nature of the QDs because ALA did not cause any reduction in cell viability (Figure S13b).

### In Vitro Cell Uptake and Optical Detection

**Figure 4:**
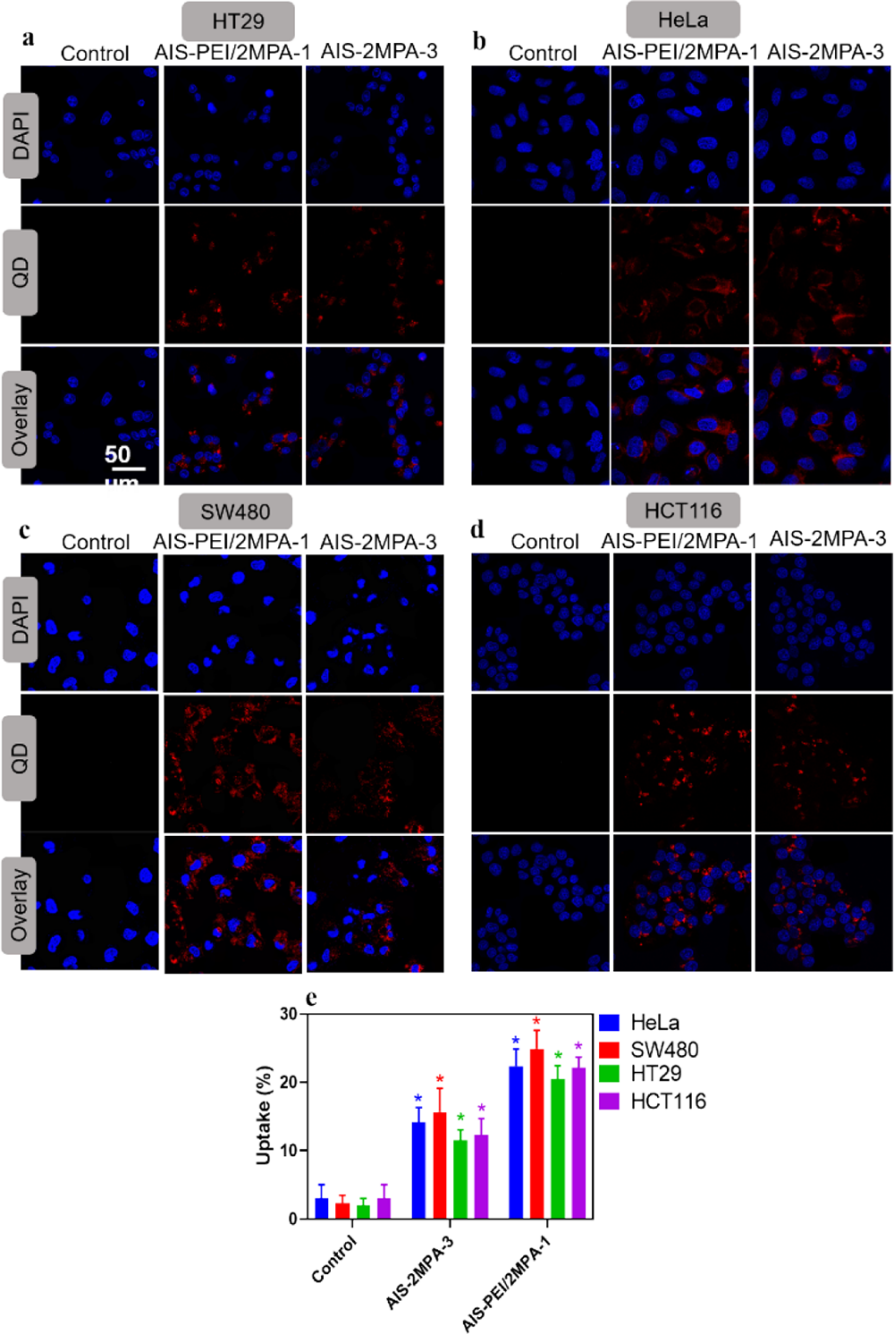
Cellular localization of the control cells, AIS-PEI/2MPA-1, and AIS-2MPA-3 QDs by (a) HT29, (b) HeLa, (c) SW480, and (d) HCT116 cells. The samples at 2 μg/mL cation concentration were incubated with cells for 24 h in 2D culture. Red: luminescence from the QDs (excitation/emission:488/580–780 nm), blue: nuclear stain. (e) Intracellular quantification of AIS-PEI/2MPA-1 and AIS-2MPA-3 QDs in HeLa, SW480, HT29, and HCT116 cell lines in 2D culture. Measurements were done by an ICP-MS instrument. The samples at 2 μg/mL cation concentration were incubated with cells for 24 h. The data are expressed as mean ± S.D. using two-way ANOVA with Tukey’s multiple comparison test (*n* = 3), (*p* < 0.05).

HT29, HeLa, SW480, and HCT116 cells treated with 2 μg/mL [cation] of AIS-2MPA-3 and AIS-PEI/2MPA-1 QDs for 24 h exhibited intense intracellular optical signals, indicating effective internalization of both QDs to each cell line (Figure 4a–d). The intensity and clarity of the intracellular signal support the potential of these QDs for optical–medical imaging. There seems to be more luminescence in the cells treated with the cationic QDs. Hence, to quantify the uptake of QDs by these cell lines, the concentration of Ag and In in the QD-treated cells was determined by ICP-MS. After 24 h incubation with QDs (2 μg/mL), about 20–25% of the AIS-PEI/2MPA-1 and 10–15% of the AIS-2MPA-3 QDs were internalized by the different cell lines according to the ICP-MS analysis of the lysed cells with no significant difference between different cell lines (Figure 4e). In addition to the more toxic nature of PEI than 2MPA, this higher uptake of the cationic QDs may also contribute to the higher toxicity of the AIS-PEI/2MPA-1 QDs.

Fluorescence microscopy was also conducted on the 3D spheroids of HT29 and SW480 cells with AIS-PEI/2MPA-1 (2 μg/mL) and AIS-2MPA-3 QDs (2 and 50 μg/mL) and their ALA-loaded formulations (Figure S14a,b). Both QDs showed strong luminescence, and in the anionic ones, the optical signal seemed to be stronger as the concentration increased. The ALA-loaded versions seem to provide more signal, especially in SW480, probably due to PpIX generation with luminescence at around the same wavelengths as these AIS QDs. Overall, the bright red fluorescence observed in the 3D matrix originates from the embedded cells rather than the collagen matrix. This strong AIS-based intracellular luminesce observed within the 3D matrix further supports the candidacy of these QDs for optical imaging and/or utilization of such nanoparticles for image-guided therapy.

### In Vitro PDT Studies

ALA can act as a prodrug of a strong photosensitizer, PpIX, which produces highly toxic ROS upon irradiation, causing apoptosis/necrosis. After the internalization of the ALA conjugates by the cells, ALA was converted to PpIX via the heme-synthesis pathway.^77^ Therefore, delivery of ALA into the cells in a therapeutic dose, its release, and its intracellular conversion to PpIX in the cancer cells are crucial steps for effective PDT. The fluorescence of PpIX at 635 nm can be used to determine the intracellular PpIX amount. However, because QDs have absorbance at an excitation wavelength of 420 nm and luminescence at the same region with PpIX, it interfered with the measurements (Figure S15). Yet, an indication of higher PpIX generation with the QD-loaded ALA was observed in addition to a slight dose and cell type dependence mostly at high concentrations. The differences between the generated PpIX in the cell lines are due to the various ALA metabolisms and conversion of ALA to PpIX by different mitochondrial enzymes.^76^

**Figure 5:**
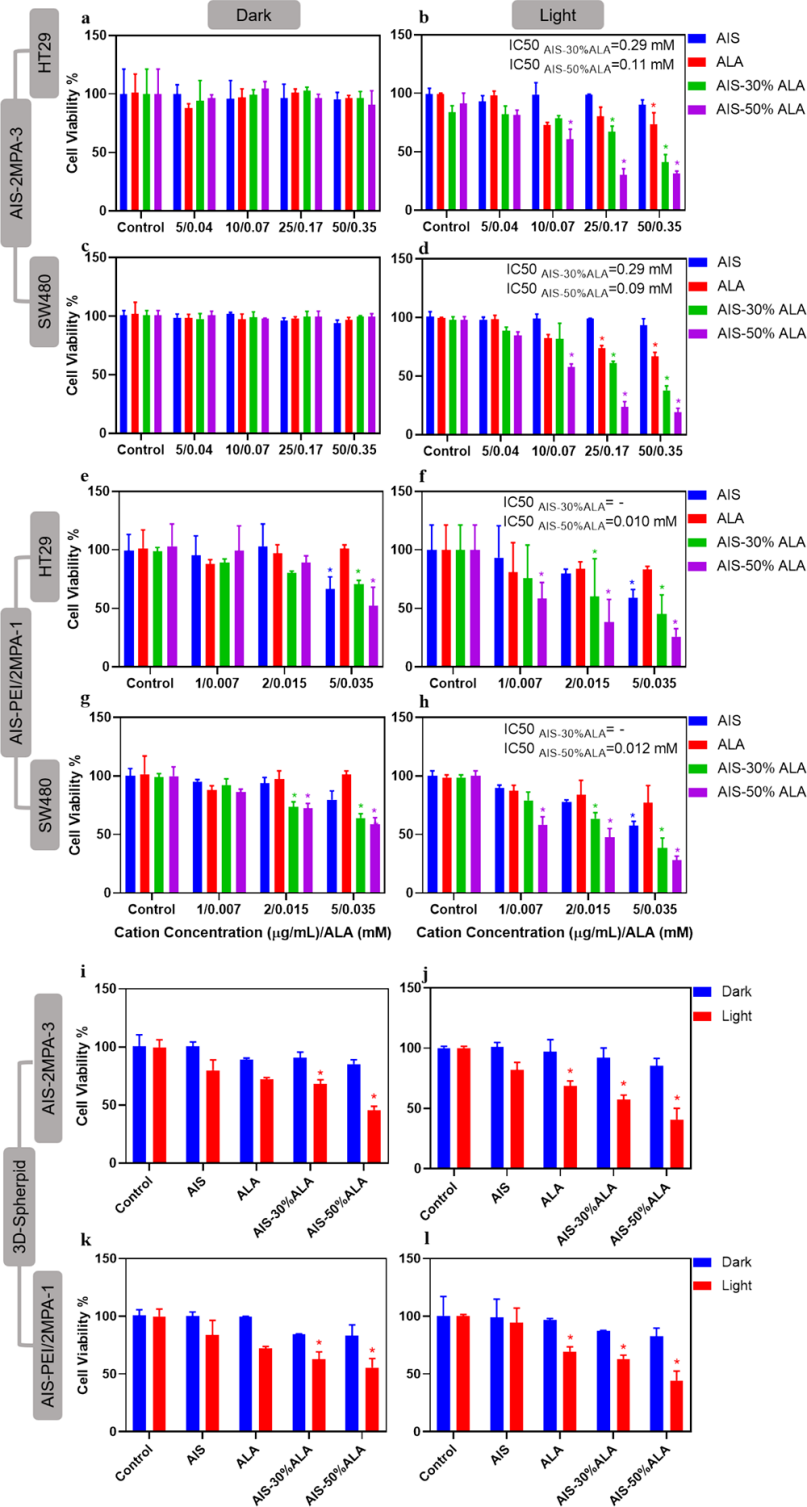
Viability of (a,b) HT29 and (c), (d) SW480 cells treated with AIS-2MPA-3, AIS-2MPA-3-30%ALA, and AIS-2MPA-3-50%ALA QDs and (e,f) HT29 and (g,h) SW480 cells treated with AIS-PEI/2MPA-1, AIS-PEI/2MPA-1-30%ALA, and AIS-PEI/2MPA-1-50%ALA QDs after 24 h incubation with and without PDT measured by Alamar blue compared to the untreated cells. Viability of 0.35 mM ALA of AIS-2MPA-3, AIS-2MPA-3-30%ALA, and AIS-2MPA-3-50%ALA QDs on (i) HT29 and (j) SW480 3D-spheroid models and 0.015 mM ALA of AIS-PEI/2MPA-1, AIS-PEI/2MPA-1-30%ALA, and AIS-PEI/2MPA-1-50%ALA on (k) HT29 and (l) SW480 3D-spheroid models treated with QDs and illuminated at 420 nm blue light for 5 min. PDT control indicated cells treated only with 420 nm irradiation for 5 min. The data are expressed as mean ± S.D. using two-way ANOVA with Tukey’s multiple comparison test (*n* = 3), (*p* < 0.05).

In vitro PDT studies were performed on the 2D and 3D cell cultures using blue light (420 nm, 7 mW/cm^2^) for 5 min. Because MTT is not suitable to check viability in 3D cell cultures, Alamar blue was used to determine the viability of cells before and after PDT (Figure 5). The viabilities of the 2D-cell cultures after PDT were also tested with the MTT assay (Figure S16). All doses were based on ALA in Figure 5 because it is critical to compare the phototoxicity of free ALA versus QD-delivered ALA. The corresponding QD concentrations are given in Table S2. All PDT studies were performed on two colorectal cancer cell lines: SW480 and HT29.

Anionic AIS-2MPA-3 and its ALA-loaded versions showed no dark toxicity in the studied range (Figure 5a,c). ALA created effective toxicity upon irradiation only at the highest dose, 0.35 mM in the HT29 cell line, and at and above 0.17 mM in the SW480 cell line. However, the ALA-loaded AIS-2MPA-3 QDs induced greater phototoxicity in a dose- and ALA loading-dependent manner. The viability of the HT29 cells dropped to ∼40% (30% ALA loading) and ∼30% (50% ALA loading) at 0.35 mM ALA, respectively, whereas free ALA provided 75% viability at this dose. The effect was more dramatic on the SW480 cells with ∼40% (30% ALA loading) and ∼20% (50% ALA loading) viability, whereas free ALA provided ∼70% viability at 0.35 mM ALA. Because no phototoxicity was detected in the cells treated with AIS-2MPA-3, enhanced phototoxicity should indicate better ALA-PDT when delivered with these nanoparticles (Figure 5b,d).

AIS-2MPA-3-30%ALA provided IC50 at 0.29 mM and 0.25 mM in HT29 and SW480 cells. This was improved by using AIS-2MPA-3-50%ALA QDs: IC50 was 0.11 and 0.09 mM for HT29 and SW480 cells, respectively. Our recent study has shown about 60 and 40% reduction in viability of SW480 and HT29 cell lines treated with ALA-loaded (0.35 mM [ALA]) anionic Ag_2_S QDs after 5 min irradiation at 420 nm.^76^ The higher sensitivity of SW480 cells to ALA-PDT is at least partially due to the significantly better conversion of ALA to PpIX by the SW80 cells.^76^ The stronger phototoxicity observed here at the same ALA dose may be due to the compositional difference of the carrier QD.

Cationic AIS-PEI/2MPA QDs and their ALA-loaded versions were tested between 0.007 and 0.015 mM ALA doses (0.6–2.24 μg/mL [cation]) due to the QD-based dark toxicity at higher concentrations (Figure S12). At these concentrations, ALA or the cationic AIS QDs showed no dark toxicity in either cell line (Figure 5e,g). Significant dark toxicity was only recorded at 0.015 mM ALA in the SW480 cell line. Irradiation of the AIS-PEI/2MPA-1-treated cells did not cause a significant change in the viability with respect to the dark toxicity of these cationic QDs. At these concentrations, ALA did not cause notable phototoxicity either. However, phototoxicity of ALA delivered by the AIS-PEI/2MPA-50%ALA QDs effectively reduced the viability even at the 0.007 mM ALA dose to 57 and 58% in the HT29 and SW480 cell lines, respectively (Figure 5f,h). At 0.015 mM ALA concentration, the viability of the HT29 cells were 60 and 40% when treated with the AIS-PEI/2MPA-1-30%ALA and AIS-PEI/2MPA-1-50%ALA QDs, respectively. The SW480 cell line showed a similar trend with ∼65 and 45% viability after treatment with the AIS-PEI/2MPA-1-30%ALA and AIS-PEI/2MPA-1-50%ALA QDs. The viability of ALA loaded to the cationic PEG–chitosan nanoparticles in the study of Chung et al. reduced the viability of the CT26 cells to 40% at 0.1 mM [ALA] (1.8 J/cm^2^) while we reached the same viability after PDT at 0.015 mM [ALA] AIS-PEI/2MPA-1-50%ALA QDs.^46^

All of the further in vitro experiments were performed at 0.35 mM ALA and corresponding concentrations of the AIS-2MPA-3 QDs as well as at 0.015 mM ALA and the corresponding amounts of the AIS-PEI/2MPA-1 QDs because at these doses, the 2D experiments indicated no dark toxicity but significant phototoxicity on both cell lines (Figure 5i–l).

PDT was also tested on the 3D spheroid constructs of the HT29 and SW480 cells treated with ALA, AIS QDs, and their ALA-loaded formulations. Free ALA (0.015 and 0.35 mM) reduced the viability to ∼70–75% in both the HT29 and SW480 cells after irradiation. Both QDs improved the ALA-based PDT: The viability of the cells treated with the AIS-2MPA-3-50%ALA QDs (0.35 mM ALA) reduced the viability of the HT29 cells to 45% and SW480 cells to 40% (Figure 5i,j). In the cationic AIS-PEI/2MPA-3-50%ALA QDs, irradiation of the treated spheroids reduced the viability of the HT29 and SW480 cells to 55 and 45%, respectively, at 0.015 mM ALA concentration (Figure 5k,l).

Because irradiation of free ALA and free QDs did not create significant phototoxicity and the ALA-loaded ones showed dose-dependent toxicity, ROS generation due to irradiation of the intracellular PpIX should be the primary factor responsible for the reduction of cell viability. To determine the amount of ROS generated after blue light illumination, a cell-permeable nonfluorescent dye DCFDA is utilized as the ROS indicator. The ROS oxidizes the dye to green fluorescent dichlorofluorescein (DCF). After incubation of the HT29 and SW480 cells with ALA, QDs, and ALA-loaded QDs, the production of ROS was followed by the green fluorescence intensity at 538 nm (Figure S17a,b). QD irradiation did not generate any ROS, which supports no phototoxicity observation in the absence of ALA. The ALA-loaded QDs showed significantly more ROS generation than free ALA in both cell lines, especially with 50% ALA loading to QDs. The amounts of the ROS generated by ALA-loaded cationic versus ALA-loaded anionic QDs are not significantly different, although the anionic ones carried more ALA at the studied doses of the two QDs (0.0.015 versus 0.35 mM ALA). The release of ALA from the cationic QDs after 24 h was only about 10% more than the ALA released from the anionic QDs (Figure S11c,d). At especially low ALA concentrations, the amount of PpIX produced from 0.015 and 0.35 mM ALA is similar (Figure S15). These two data support a similar amount of ROS generated by ALA delivered by either delivery vehicle upon irradiation. More toxicity via the cationic delivery vehicle was observed when the phototoxicity of the ALA-loaded QDs is at similar ALA concentrations, but the cationic versus anionic QD delivery was considered. For example, at 0.07 mM ALA delivered by cationic AIS-PEI/2MPA-1-50%ALA versus anionic AIS-2MPA-3-50%ALA, the viabilities of both cells were around 15–30% versus 20–30%, respectively. Considering no dark toxicity of the anionic QD but significant dose-dependent cytotoxicity of the cationic QD, the anionic AIS-2MPA QDs are better delivery vehicles when the above discussion is also taken into account.

**Figure 6:**
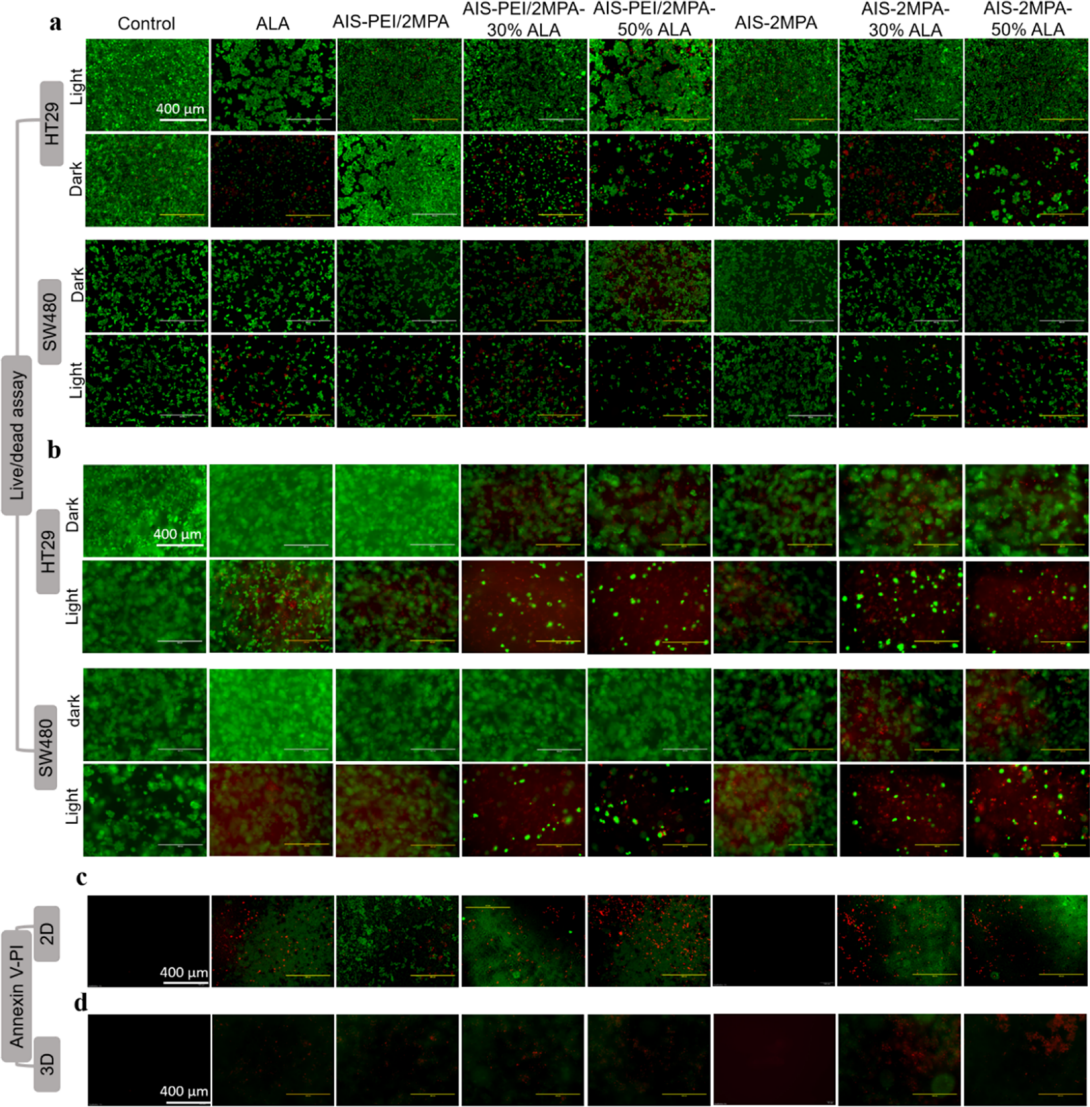
Live/dead images of HT29 and SW480 (a) 2D and (b) 3D-spheroid cultures. The cultures were treated with free ALA (0.35 mM), AIS-2MPA-3, AIS-2MPA-3-30%ALA, and AIS-2MPA-3-50%ALA QDs at 50 μg/mL and free ALA (0.015 mM), AIS-PEI/2MPA-1, AIS-PEI/2MPA-1-30%ALA, and AIS-PEI/2MPA-1-50%ALA at 2 μg/mL after 24 h incubation with/without PDT illuminated at 420 nm with a blue lamp for 5 min. Apoptotic and necrotic-induced cell death in (c) the 2D and (d) 3D cultures of the SW480 cells. Comparison of the cell death pathways induced after PDT (420 nm with a blue lamp for 5 min) with free ALA (0.35 mM), AIS-2MPA-3, AIS-2MPA-3-30%ALA, and AIS-2MPA-3-50%ALA QDs at 50 μg/mL and free ALA (0.015 mM), AIS-PEI/2MPA-1, AIS-PEI/2MPA-1-30%ALA, and AIS-PEI/2MPA-1-50%ALA at 2 μg/mL. Annexin-V was used to indicate apoptosis (green), and propidium iodide was used to indicate necrosis (red). “Control” indicates the untreated cells. Scale bars for the images are 400 μm.

To further confirm the therapeutic efficiency of the nanoparticles and PDT-dependent cell death, a calcein-AM/propidium iodide (PI) staining was used. No dead cells were seen in the absence of laser treatment at the studied doses of QDs and ALA. Treatment with the cationic or anionic AIS QDs followed by irradiation did not cause any cell death either. Few dead cells were observed when treated with the ALA-loaded cationic AIS QDs only in the SW480 cell line but not with the ALA-loaded anionic AIS QDs. However, the ALA-loaded cationic and anionic AIS QDs exhibit significantly higher cell death after irradiation both in the 2D cell cultures and 3D spheroid constructs of both cell lines. Figure 6a,b clearly shows more cell death when the AIS-2MPA-3-50%ALA QDs were used, consistent with the cell viability results and measured ROS levels.

Apoptotic/necrotic cell death caused by the PDT treatment of the cells was also determined. The SW480 cells in the 2D and 3D models were stained with Annexin-V to detect apoptosis (shown in green) and PI for detection of necrosis (shown in red) 24 h after illumination. ALA showed apoptotic cell death in 2D and a combination of apoptotic and necrotic cell death in 3D. Dramatic enhancement in the apoptotic cell population was observed with the ALA-loaded AIS QDs both in the 2D and 3D cell cultures (Figure 6c,d). QDs with 50% ALA loading caused a higher population of necrotic/apoptotic cells than the 30% ALA-loaded ones. Necrotic/apoptotic cells were more visible in the 3D spheroid and more with the cationic nanoparticles (Figure 6d).

## Conclusions

A portfolio of the emission tunable, cationic, and anionic AIS QDs was successfully prepared using a one-step, RT, aqueous synthesis method using 2MPA or PEI/2MPA as the coating/stabilizing agents for the first time in the literature. The best precursor stoichiometry for the synthesis of both the anionic and cationic AIS QDs with small sizes (less than 8 nm) and the strongest emission at 630–617 nm with QYs 19.4% (AIS-2MPA-3) and 20.3% (AIS-PEI/2MPA-1) was determined as Ag/In/S 1/10/10 at a coating/cation ratio of 5 in the cation-rich formulations. To the best of our knowledge, these are among the highest reported QYs for AIS, which lacks the Zn dopant or ZnS shell. Moreover, these QDs showed excellent colloidal stability with enhanced emission intensity over time. Although emission tunability was observed, strong emission around 700 nm was not achieved. This may be studied further by changing the coating/cation ratio and/or PEI/2MPA ratio in the future.

The in vitro studies showed that both the AIS QDs are internalized by various cancer cells (HeLa, HCT116, SW490, and HT29) and exhibit strong intracellular luminescence above 600 nm (within the optical imaging window) upon 488 nm excitation, proving their efficiency in medical imaging. The cationic AIS QDs were internalized slightly more than the anionic ones by all cell types but were also more toxic. Because the AIS-2MPA QDs caused no significant cytotoxicity up to 100 μg/mL [cation], the AIS-PEI/2MPA QDs reduced the viability of the cells above 2 μg/mL [cation].

After electrostatic loading of ALA to the QDs at two different concentrations (30 and 50% ALA with respect to the coating amounts), both QDs released ALA preferentially in acidic pH (up to 80% after 30 h) with less than 10% release at the physiological pH. Hence, the acidic tumor microenvironment could enhance the ALA release at the tumors selectively, thereby enhancing tumor localization and subsequently enhancing the conversion of ALA to PpIX.

A dose-dependent phototoxicity surpassing the phototoxicity of free ALA was observed after PDT with a 420 nm blue lamp for 5 min in either cationic or anionic AIS QDs. Reduction in cell viability was correlated with ROS generation and apoptotic/necrotic cell death. In the 3D spheroids, while 0.35 mM free ALA reduced the viability by about 25–30% in both cells lines, the anionic AIS QDs with 50% ALA loading reduced it by about 55–60%. Similarly, 0.015 mM ALA delivered by the cationic QDs loaded with 50% ALA caused 45–55% reduction of the cell viability. In the 2D cell cultures, phototoxicity was even greater, reacting to 70–80% reduction in viability. Thus, the IC50 values of the ALA were reduced significantly when delivered with the AIS QDs.

In terms of comparing the cationic and anionic AIS as a delivery vehicle for ALA, the data suggest that they are comparable; however, intrinsically higher toxicity of the cationic one (due to the presence of PEI) requires lower doses, which may be even advantageous if, for example, coupled with molecular targeting of the tumors in the future. Alternatively, anionic AIS appear to offer a safe delivery vehicle.

In the clinical scenario, the tumor selectivity of such multimodal theranostic nanoparticles will also be aided by the EPR effect, which promotes the accumulation of the nanoparticles and hence ALA at the tumor site. This would lead to the enhanced release and conversion of ALA to PpIX in the tumor cells owing to the faster release of ALA at the acidic tumor microenvironment coupled with higher conversion of ALA to PpIX in the tumor cells, thereby inducing higher phototoxicity to the tumor cells when irradiated. In addition, another level of tumor selectivity may be achieved by tagging these QDs with tumor-specific ligands, such as folic acid, anti-Her2, or anti-EGFR antibodies.^3,77,78^

Furthermore, considering the benefits observed in combination therapies, the AIS QDs loaded with ALA and an antibiotic or anti-cancer drug may dramatically enhance the treatment of such difficult to treat diseases.^77^ These cationic and anionic AIS QDs loaded with ALA or other PDT agents also have significant potential in antibacterial/antiviral PDT.

Hence, these cationic and anionic AIS QDs with strong and stable luminescence above 600 nm with strong intracellular optical signals to provide image-guided therapy and capacity to deliver the therapeutic agents emerge as easy to make promising theranostic nanoparticles.

## Supporting Information Available

Absorbance and emission spectra of AIS QDs, TEM images, Tauc plots, Urbach energy, band structures, isothermal titration curves, time and pH-dependent ALA release profiles, viability of the cancer cells after 48 h incubation with the agents and viability of the treated healthy cell line, fluorescence microscopy images of the 3D cancer cell spheroids treated with QDs, tables showing the time-dependent changes in the QD properties and summarizing the details of the QDs used in the in vitro experiments, detailed description of PpIX detection in treated cancer cells (PDF)
