# Supplementary material for: One-Step Aqueous Synthesis of Anionic and Cationic AgInS2 Quantum Dots and Their Utility in Improving the Efficacy of ALA-Based Photodynamic Therapy
Source: Inorg Chem. 2022 Feb 1;61(6):2846–63. doi: 10.1021/acs.inorgchem.1c03298 (PMC8895404; doi:10.1021/acs.inorgchem.1c03298)
Supplement: Supplementary file 1 — ic1c03298_si_001.pdf [file ic1c03298_si_001.pdf]

Supporting Information:

One-step aqueous synthesis of anionic and cationic $AgInS_2$ quantum dots and their utility in improving the efficacy of ALA-based photodynamic therapy

Mahshid Hashemkhani[1], Marilena Loizidou[2], Alexander J. MacRobert[2], Havva Yağci Acar*[1, 3, 4]

[1]Koc University, Graduate School of Materials Science and Engineering, Rumelifeneri Yolu, Sariyer

34450, Istanbul, Turkey.

[2]Division of Surgery and Interventional Science, Centre for Nanomedicine and Surgical Theranostics, University College London, Royal Free Campus, Rowland Hill St, London NW3 2PE

[3]Koc University, Department of Chemistry, KUYTAM, Rumelifeneri Yolu, Sariyer 34450, Istanbul, Turkey

**Corresponding Authors:*

Havva Yagci Acar

Address: Department of Chemistry, Koc University, Rumelifeneri Yolu, Sariyer 34450, Istanbul, Turkey

E-mail: fyagci@ku.edu.tr; Fax: +902123381559; Tel: +902123381742

Alexander J. MacRobert

Address: Division of Surgery and Interventional Science, Centre for Nanomedicine and Surgical Theranostics, University College London, Royal Free Campus, Rowland Hill St, London NW3 2PE

E-mail: a.macrobert@ucl.ac.uk

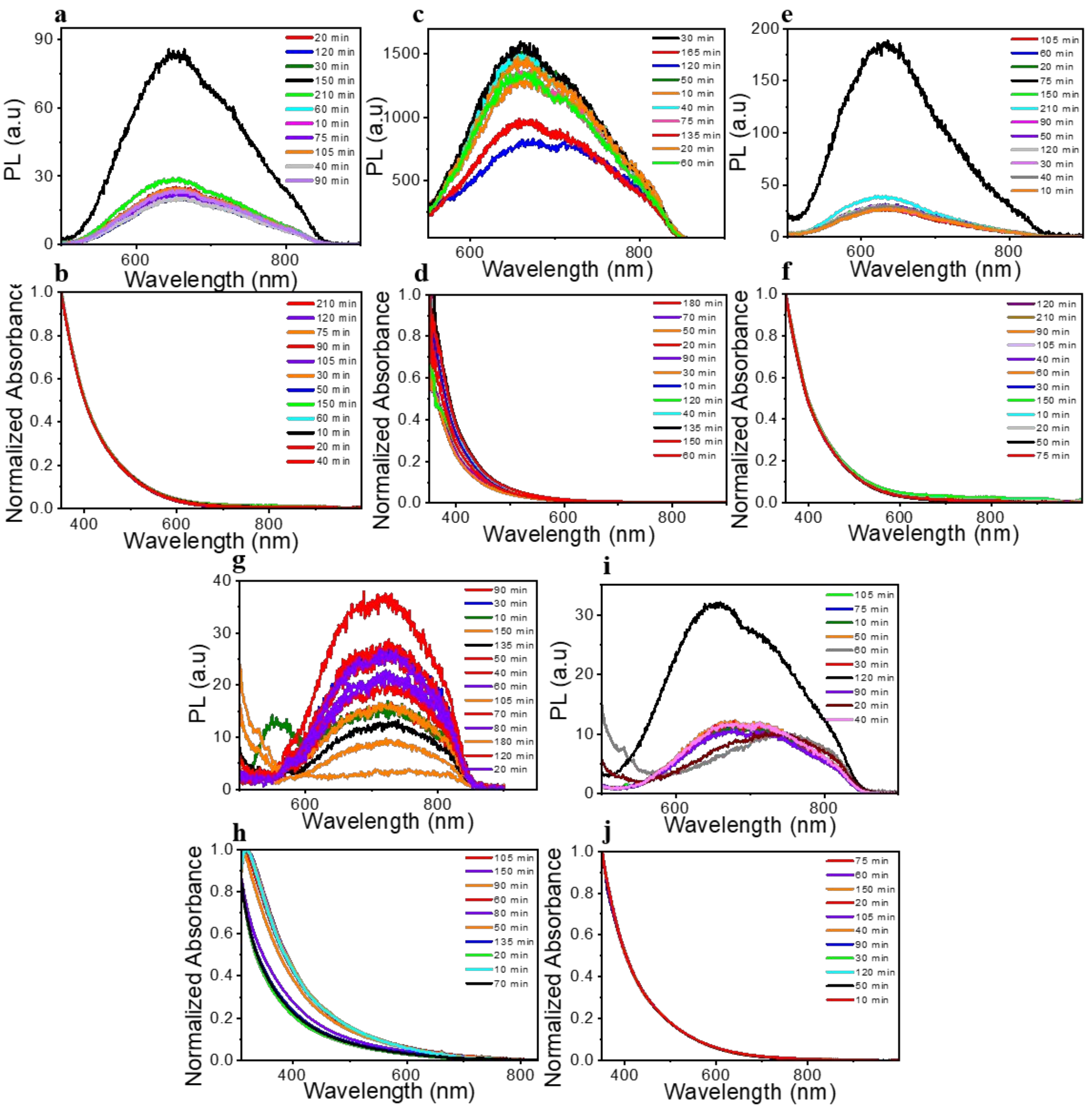

Figure S1: Time dependent changes in the photoluminescence spectra and UV-vis spectra of (a-b) AIS-2MPA-1, (c-d) AIS-2MPA-2, (e-f) AIS-2MPA-3, (g-h) AIS-2MPA-4 and (i-j) AIS-2MPA-5 during the synthesis.

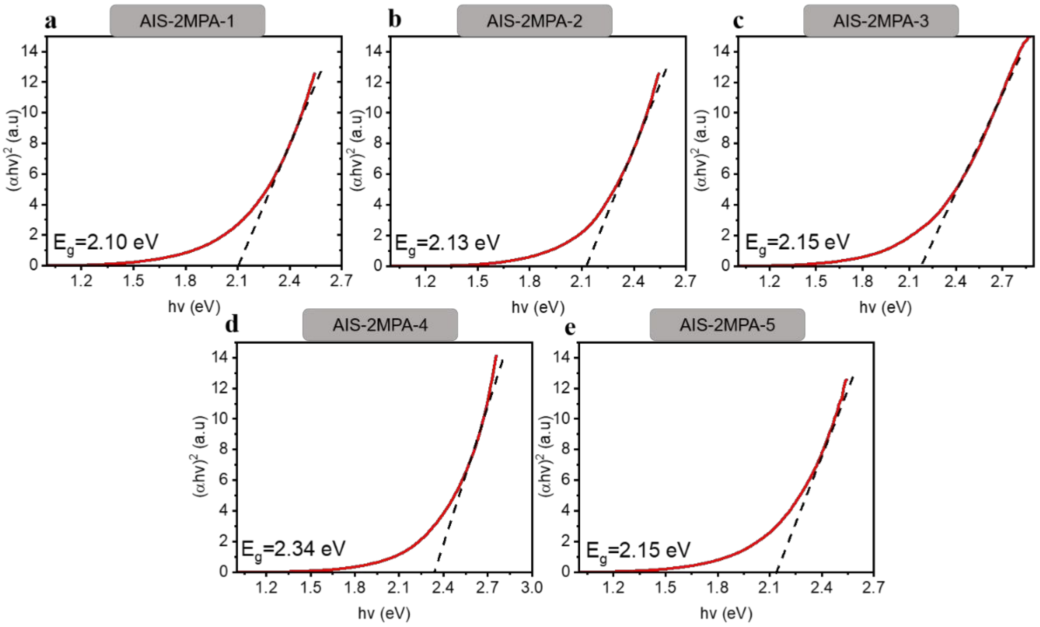

Figure S2: Tauc plots for (a) AIS-2MPA-1, (b) AIS-2MPA-2, (c) AIS-2MPA-3, (d) AIS-2MPA-4 and (e) AIS-2MPA-5 QDs.

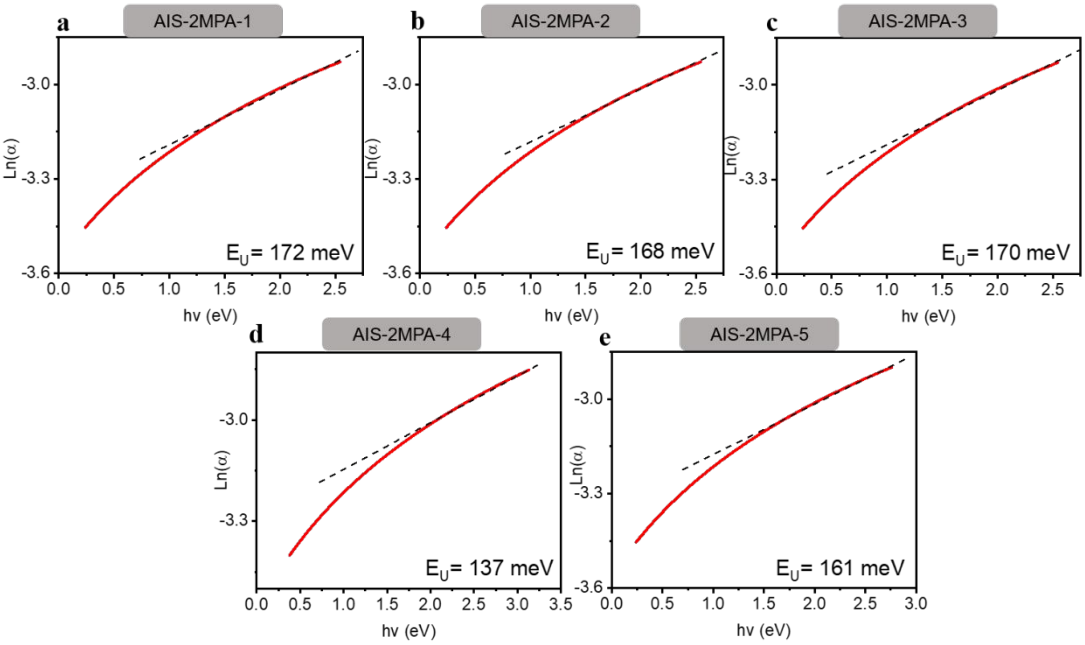

Figure S3: Urbach energies of (a) AIS-2MPA-1, (b) AIS-2MPA-2, (c) AIS-2MPA-3, (d) AIS-2MPA-4 and (e) AIS-2MPA-5 QDs.

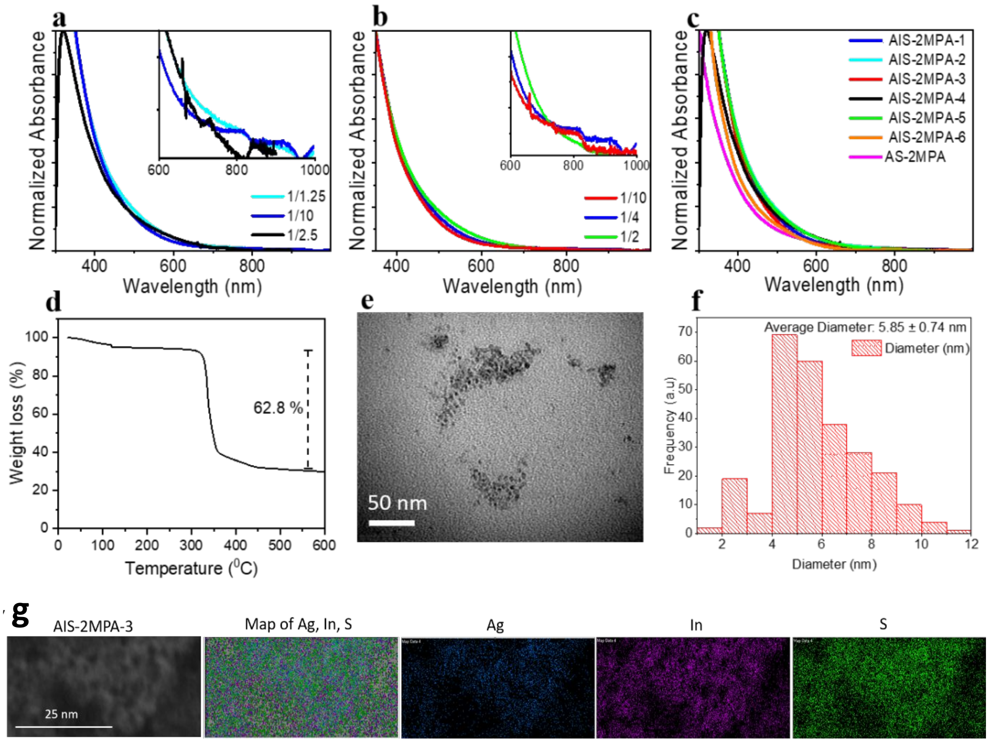

Figure S4: (a) The UV-vis spectra of AIS-2MPA QDs prepared at a constant molar ratio of Ag/In: 1/4, cations/coating: 1/5 and different molar ratios of Ag/S. (b) The UV-vis spectra of the AIS-2MPA QDs prepared at a constant molar ratio of Ag/S: 1/10, cations/coating: 1/5 and different molar ratios of Ag/In. (c) The UV-vis spectra of all AIS-2MPA QDs prepared at different conditions. (d) Thermogravimetric analysis (TGA) of AIS-2MPA-3 QDs. (e) TEM image, (f) histogram and (g) EDS analysis of AIS-2MPA-3 QDs.

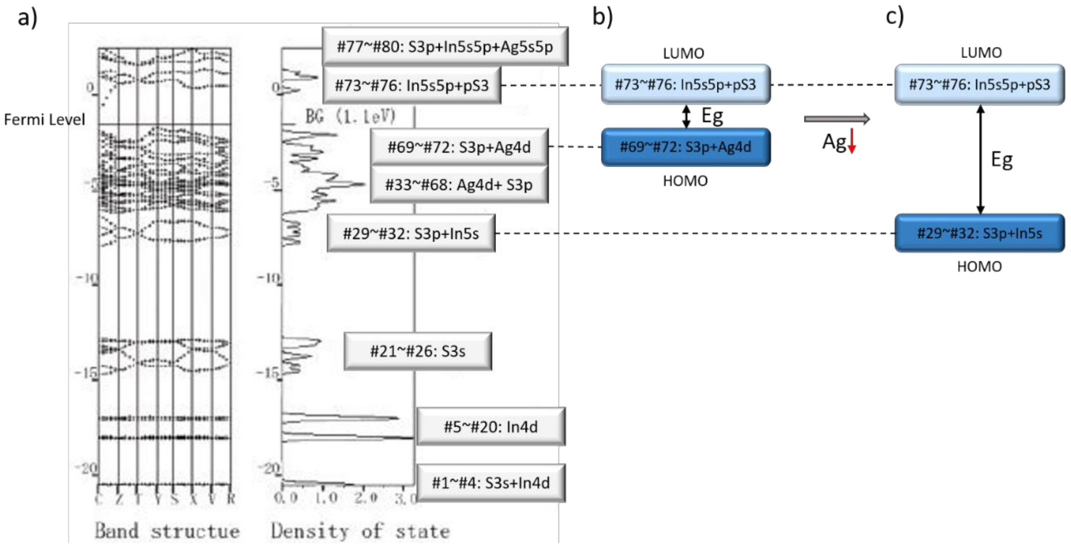

Figure S5: (a, b, c) Band structures and densities of states $AgInS_2$ calculated by the density functional method.

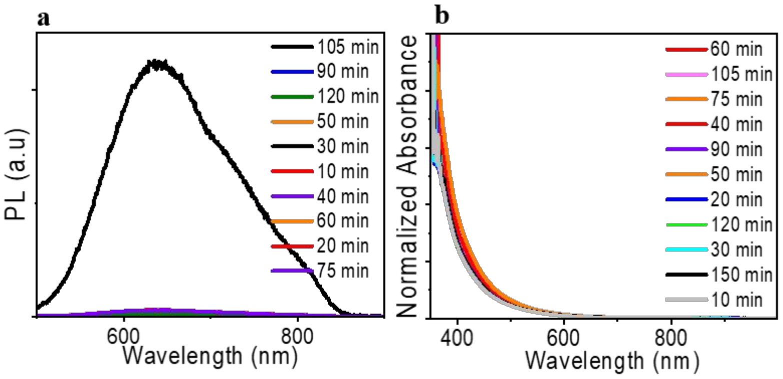

Figure S6: The (a) PL and (b) UV-vis spectra of the AIS-PEI/2MPA-2 samples withdrawn from the reaction mixture at different time points.

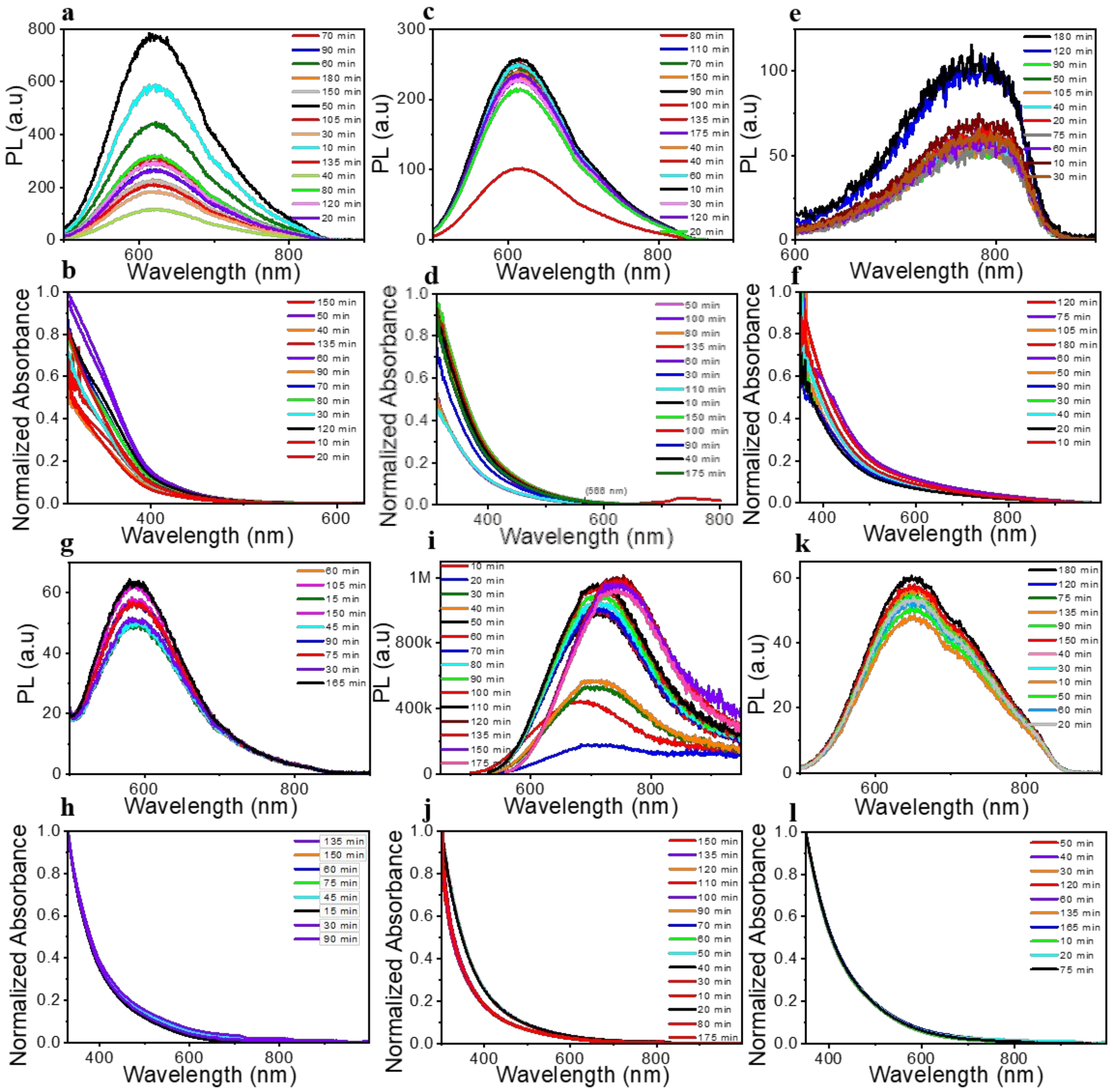

Figure S7: Time dependent changes in the PL and UV-vis spectra of (a-b) AIS-PEI/2MPA-1, (c-d) AIS-PEI/2MPA-3, (e-f) AIS-PEI/2MPA-4, (g-h) AIS-PEI/2MPA-5, (i-j) AIS-PEI/2MPA-6 and (k-l) AIS-PEI/2MPA-7 QDs during the reaction.

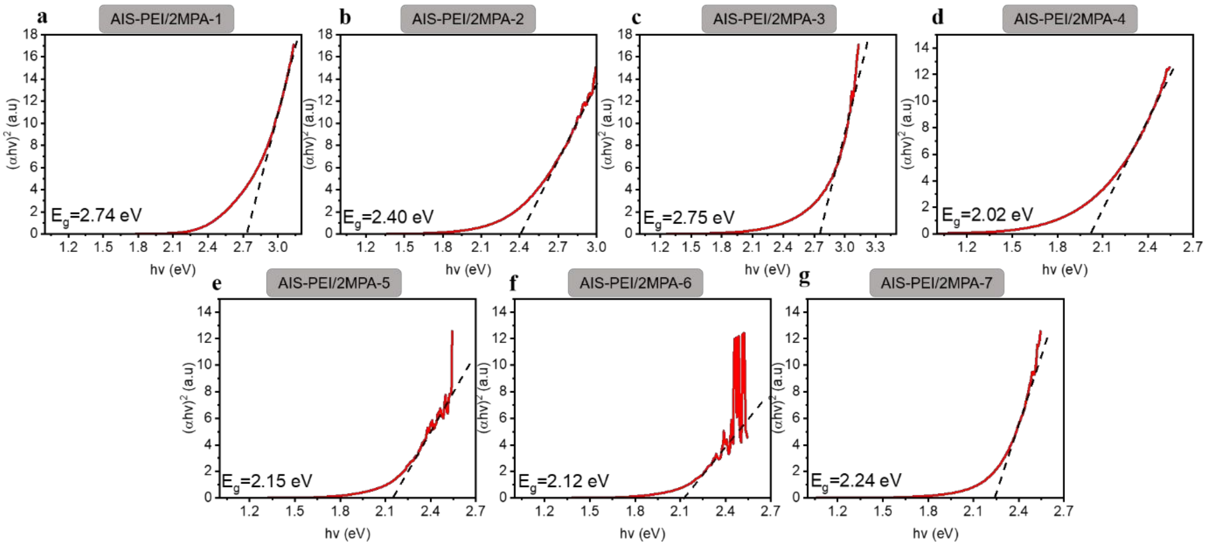

Figure S8: Tauc`s plots for (a) AIS-PEI/2MPA-1, (b) AIS- PEI/2MPA-2, (c) AIS- PEI/2MPA-3, (d) AIS- PEI/2MPA-4, (e) AIS- PEI/2MPA-5, (f) AIS- PEI/2MPA-6 and (g) AIS- PEI/2MPA-7 QDs.

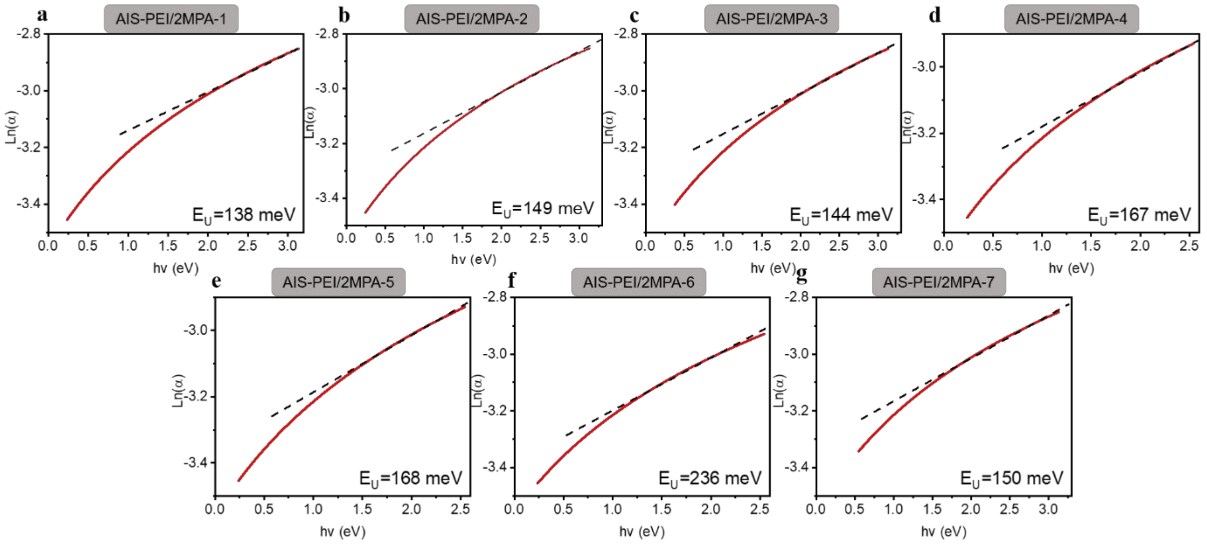

Figure S9: Urbach energies of (a) AIS-PEI/2MPA-1, (b) AIS- PEI/2MPA-2, (c) AIS-PEI/2MPA-3, (d) AIS-PEI/2MPA-4, (e) AIS-PEI/2MPA-5, (f) AIS-PEI/2MPA-6 and (g) AIS-PEI/2MPA-7 QDs.

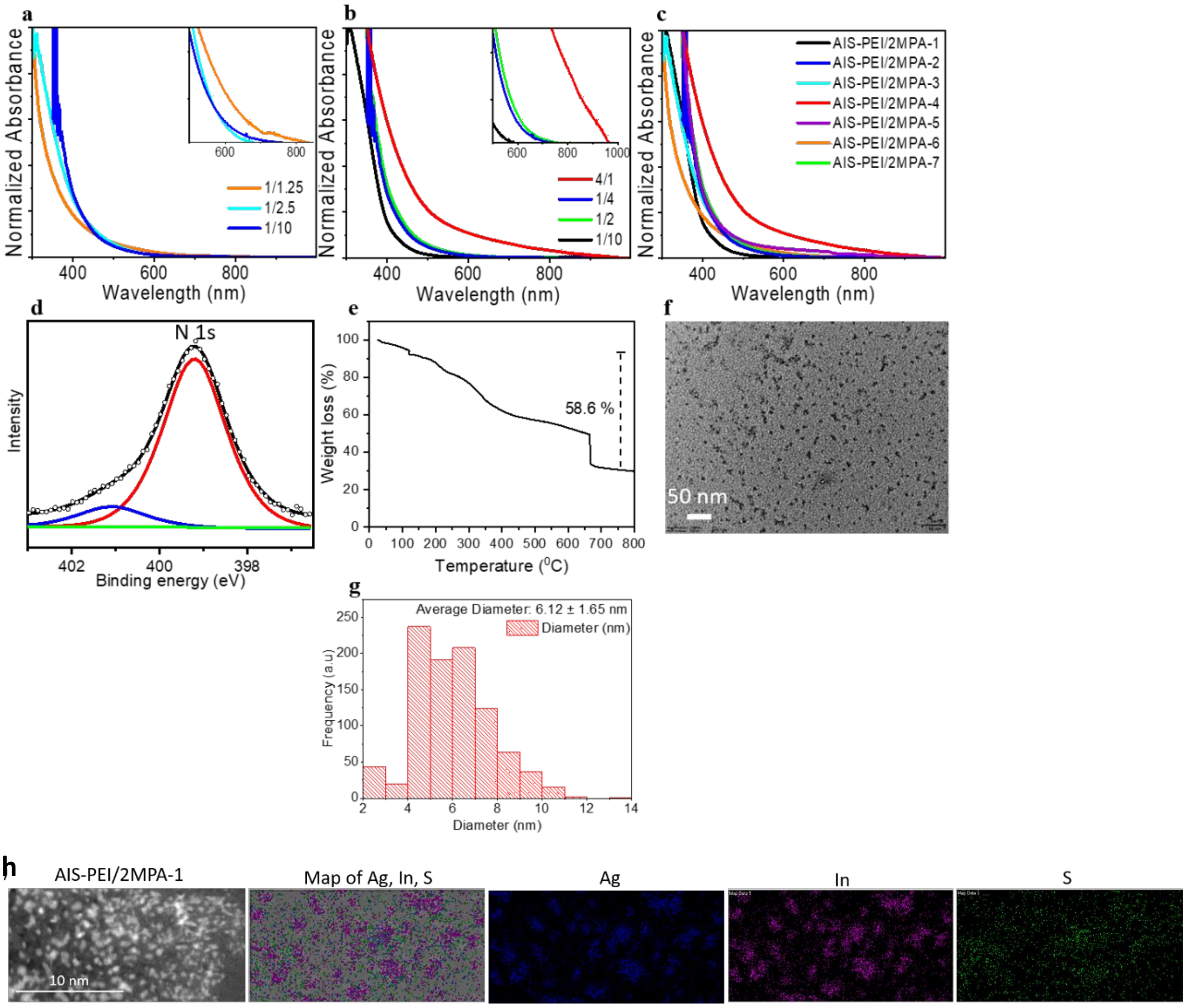

Figure S10: (a) The UV-vis spectra of AIS-PEI/2MPA QDs prepared at a constant molar ratio of Ag/In: 1/4, cations/coating: 1/5 and different molar ratios of Ag/S. (b) The UV-vis spectra of the AIS-PEI/2MPA QDs prepared at a constant molar ratio of Ag/S: 1/10, cations/coating: 1/5 and different molar ratios of Ag/In. (c) The UV-vis spectra of all AIS-PEI/2MPA QDs prepared at different conditions. (d) XPS spectra of N 1s for AIS-PEI/2MPA-1 QDs. (e) Thermogravimetric analysis (TGA) of AIS-PEI/2MPA-1 QDs. (f) TEM image, (g) histogram and (h) EDS analysis of AIS-PEI/2MPA-1 QDs.

Table S1: Time dependent changes in particle properties [a]

	λ_{emi} [a] (nm)	Dh-number [b] (nm)	Dh-intensity [c] (nm)	Zeta potential (mv)
**AIS-2MPA-3-1st day**	634	5.2±1.1	38.2±11.2	-42.1±1.9
**AIS-2MPA-3-1st month**	627	3.9±0.5	29.9±2.1	-38.1±3.1
**AIS-2MPA-3-2nd month**	617	4.3±0.6	62.6±9.2	-39.4±3.1
**AIS-2MPA-3-3rd month**	612	4.9±1	38.7±8.6	-44.6±1.3
**AIS-PEI/2MPA-1-1st day**	617	7.7±0.9	27.9±4.1	10.7±2.1
**AIS-PEI/2MPA-1-1st month**	603	8.9±2.1	38.9±7.2	9.9±4.2
**AIS-PEI/2MPA-1-2nd month**	620	8.3±1.4	55.4±8.7	9.5±1.7
**AIS-PEI/2MPA-1-3rd month**	593	9.2±3.3	38.1±14.1	11.8±1.9

These QDs were synthesized according to recipes of AIS-2MPA-2 and AIS-PEI/2MPA-1; therefore, particle properties are slightly different than those reported in Table 1 and 2. [a] Emission maxima. [b] Hydrodynamic diameter measured by DLS and reported as the number average. [c] Hydrodynamic diameter measured by DLS and reported as the intensity average.

Table S2: Composition and properties of ALA loaded AIS-2MPA-3 and AIS-PEI/2MPA-1 QDs used in the *in vitro* studies

Sample	PDI [a]	Dh-number [b] (nm)	Dh-intensity [c] (nm)	Zeta potential (mv)	[cation] (µg/mL)	[ALA] (mM)
**AIS-2MPA-3**	0.39	5.2±1.1	38.2±11.2	-41.1±1.9	10	-
**AIS-2MPA-3-30%ALA**	0.37	5.7±0.7	62.1±4.3	-42.7±2.8	10	0.07
**AIS-2MPA-3-50%ALA**	0.29	5.9±0.6	50.6±7.8	-43.9±1.5	6	0.07
**AIS-PEI/2MPA-1**	0.23	7.7±0.9	27.9±4.1	12.7±2.1	10	-
**AIS-PEI/2MPA-1-30%ALA**	0.21	10.1±2.1	27.4±2.8	14.1±3.8	10	0.07
**AIS-PEI/2MPA-1-50%ALA**	0.18	9.2±1.3	33.1±6.5	15.9±0.9	6	0.07

[a] Polydispersity index, [b] Hydrodynamic diameter measured by DLS and reported as the number average. [c] Hydrodynamic diameter measured by DLS and reported as the intensity average. All QDs are in HEPES at pH=7.2.

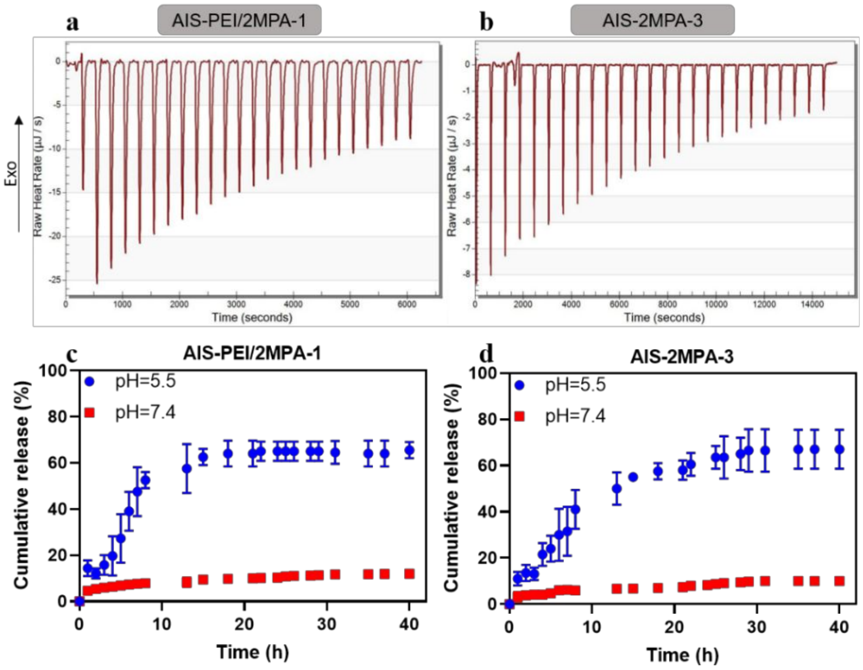

Figure S11: Isothermal titration calorimetry of (a) AIS-PEI/2MPA-1 and (b) AIS-2MPA-3 with 50 mol% ALA in HEPES at pH 7.2. In vitro release profiles of (c) AIS-PEI/2MPA-1-50%ALA and (d) AIS-2MPA-3-50%ALA in PBS solution at pH 7.4 and 5.5, 37 °C for 40 h.

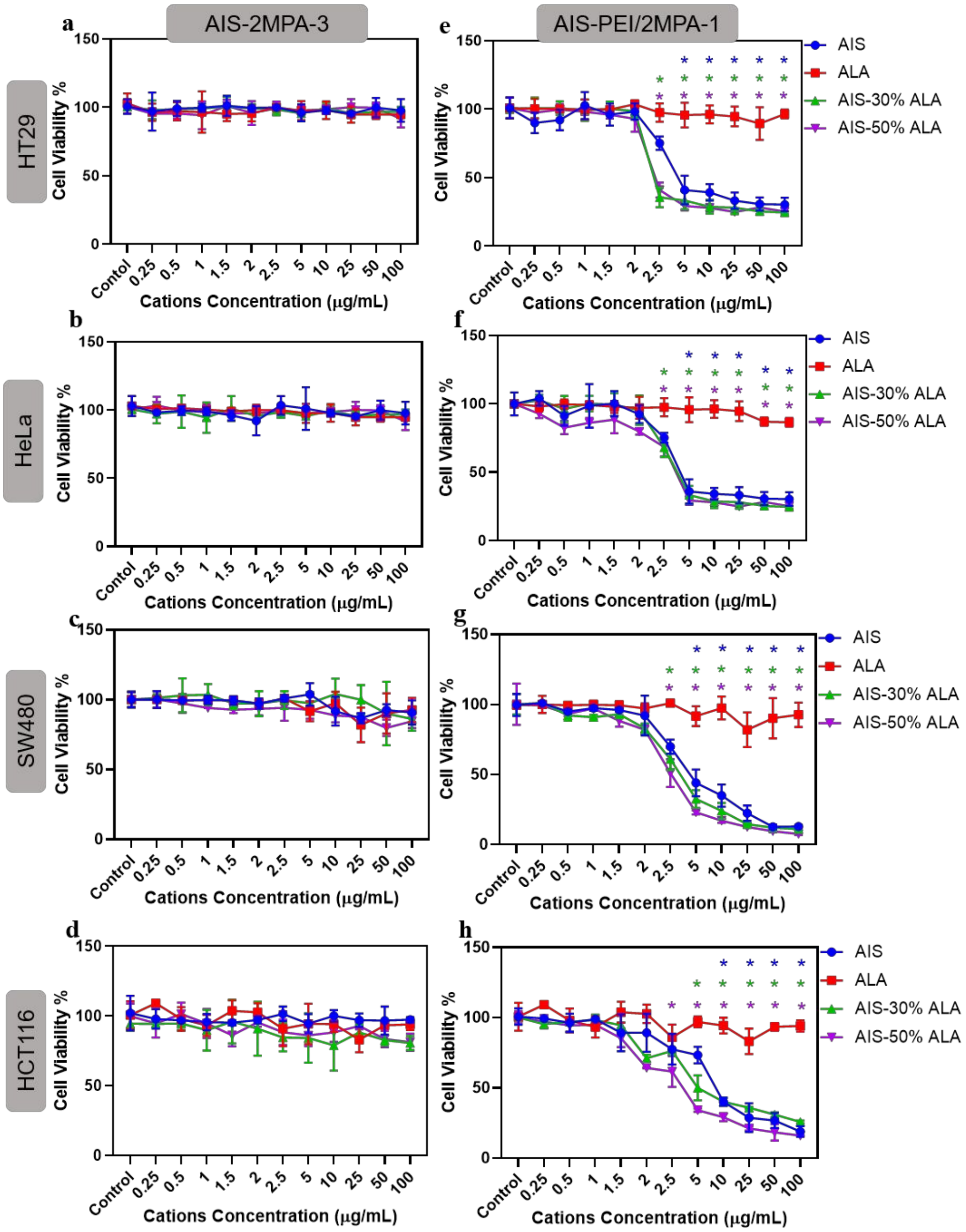

Figure S12: Viability of HT29, HeLa, SW480 and HCT116 cells treated with (a-c) AIS-2MPA-3 (d-f) AIS-PEI/2MPA-3 QDs after 48 h incubation, determined by MTT assay. Untreated cells were used as controls. The data are expressed as mean ± S.D. (n = 4). ($p<0.05$).

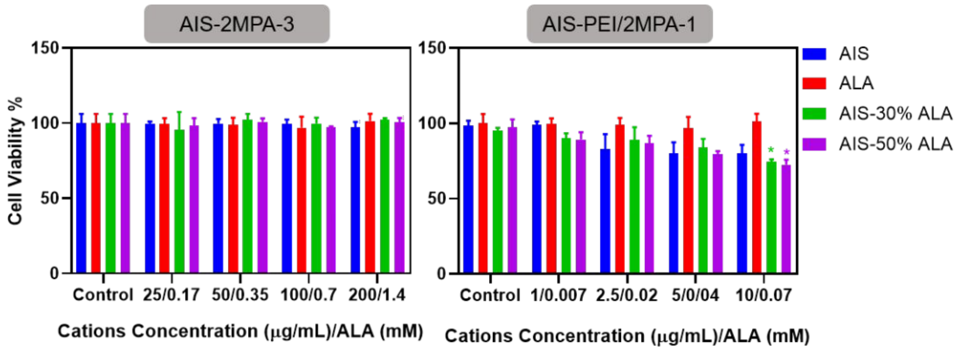

Figure S13: Viability of CCD841 cells (healthy colorectal cells) treated with (a) AIS-2MPA-3, AIS-2MPA-3-30%ALA, AIS-2MPA-3-50%ALA QDs and (b) AIS-PEI/2MPA-1, AIS-PEI/2MPA-1-30%ALA, AIS-PEI/2MPA-1-50%ALA QDs after 48 h incubation, measured by MTT assay. Untreated cells were used as control. The data are expressed as mean ± S.D. (n = 4). ($p<0.05$).

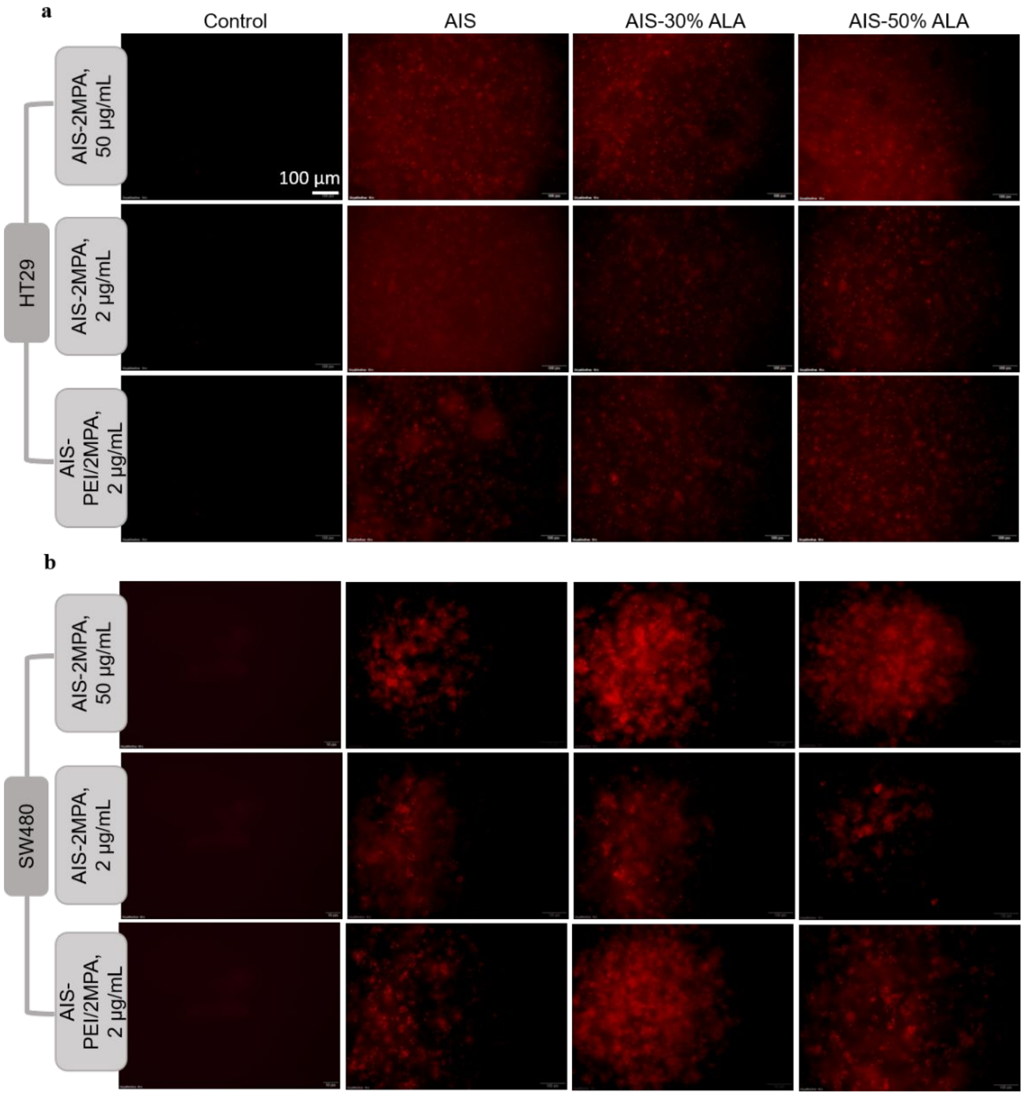

Figure S14: Cellular localization of 3D-spheroid constructs of control cells, AIS-PEI/2MPA-1 and AIS-2MPA-3 QDs by (a) HT29 and (b) SW480 cells. Excitation: 545 nm, emission 620 nm.

PpIX detection

Intracellular PpIX levels of treated HT29, HeLa, SW480 and HCT116 cells were determined from the PpIX fluorescence intensity at 635 nm with 420 nm excitation recorded on a microplate reader. However, since AIS QDs have absorbance and emission at these wavelengths, it interferes with PpIX measurements. In order to see the significance of AIS QD based delivery on the PpIX levels under these circumstances, a broad range of ALA concentration was needed; hence, the non-toxic AIS-2MPA-3 was used for a dose dependent study in the range of 0.002-0.7 mM ALA. Cells without any treatment were used as a control. Especially in SW480 cell lines, a stronger PpIX signal with 50% ALA loading to AIS QDs was clearly seen. Since the AIS content for the equivalent amount of ALA is lower when 50% ALA loading is considered compared to 30% ALA loading, QD interference with the measurement is lower (Table S2). So, especially in SW480 there is a clear advantage of delivering ALA to cells with high ALA content AIS QDs (Figure S14).

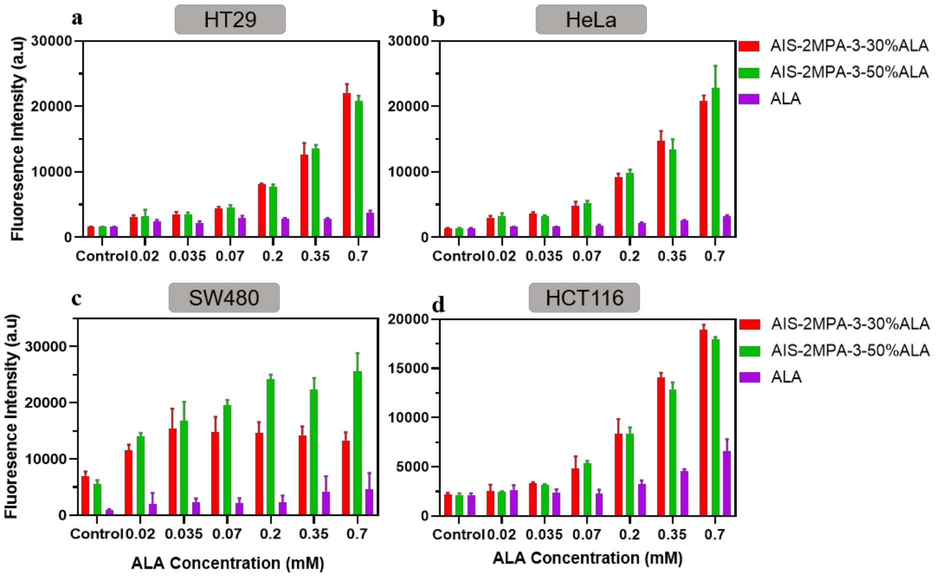

Figure S15: Intracellular PpIX levels of (a) HT29, (b) HeLa, (c) SW480 and (d) HCT116 cells after 24 h incubation with free ALA and ALA loaded AIS-2MPA-3 QDs in serum-free media. The data are expressed as mean ± S.D. (n = 4).

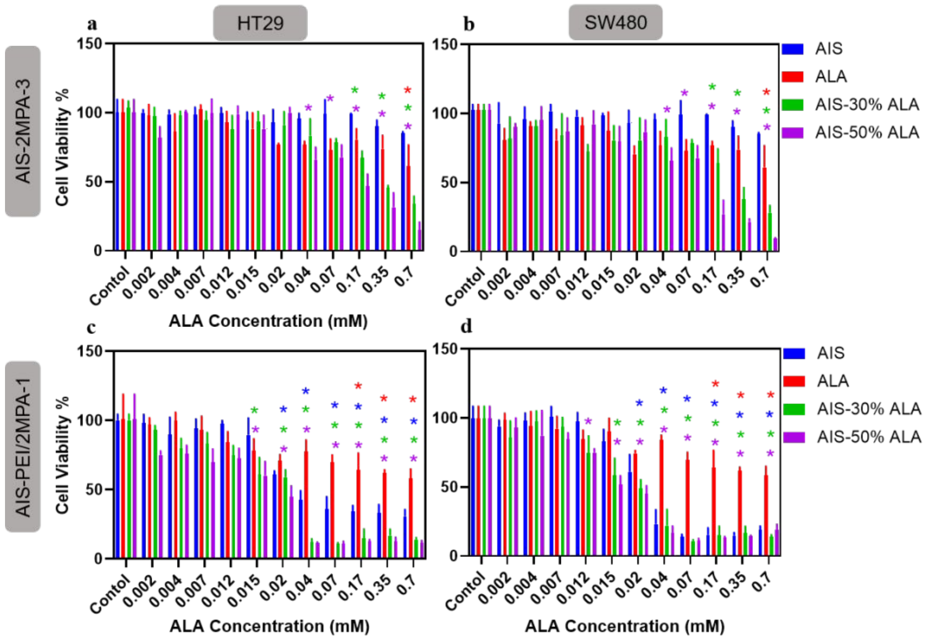

Figure S16: Viability of AIS-2MPA-3, AIS-2MPA-3-30%ALA and AIS-2MPA-3-50%ALA QDs on (a) HT29 and (b) SW480, the viability of AIS-PEI/2MPA-1, AIS-PEI/2MPA-1-30%ALA and AIS-PEI/2MPA-1-50%ALA on (c) HT29 and (d) SW480 cell lines treated with QDs for 24 h illuminated with 420 nm blue light for 5 min in 2D using MTT assay. The data are expressed as mean ± S.D. (n = 4). ($p<0.05$).

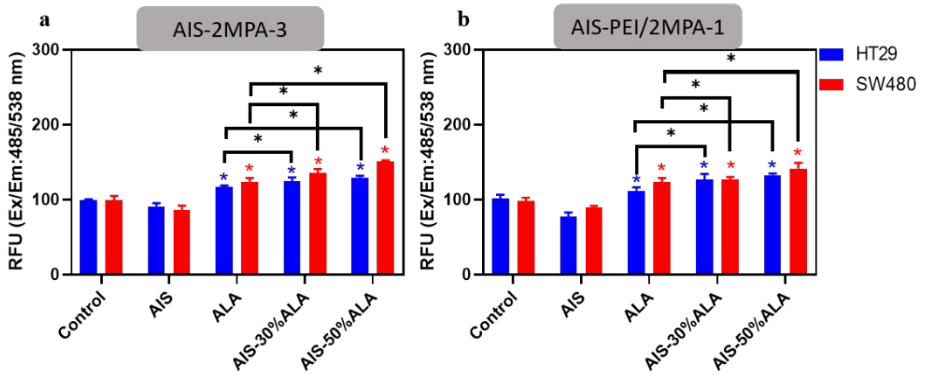

Figure S17: ROS production upon incubation with (e) AIS-2MPA-3, AIS-2MPA-3-30%ALA and AIS-2MPA-3-50%ALA QDs at 50 µg/mL and (f) AIS-PEI/2MPA-1, AIS-PEI/2MPA-1-30%ALA and AIS-PEI/2MPA-1-50%ALA after 24 h incubation at 2 µg/mL upon blue-light irradiation for 5 min. The data are expressed as mean ± S.D. (n = 3), ($p < 0.05$).
